# Causal effect of tea consumption on the increased risk of puerperal sepsis and the mediation effect of CD25 on IgD- CD38-B cell: A Mendelian randomization analysis

**DOI:** 10.1097/MD.0000000000044721

**Published:** 2025-10-24

**Authors:** Wenyu Tong, Yonghao Ouyang, Beini Zhou, Wan Peng, Hong Liu, Yanru Xiang, Jinmiao Ye, Zherui Zhang

**Affiliations:** b Research Institute of General Surgery, Jinling Hospital, Nanjing, China; a Department of Obstetrics and Gynecology, Nanjing Jianye District Maternal and Child Health Institute, Nanjing, China; c Tea Learning Major, Jiangxi Modern Polytechnic College, Nanchang, China; d Department of Infectious Diseases, The Affiliated Guangdong Second Provincial General Hospital of Jinan University, Guangzhou, China; e Department of Obstetrics and Gynecology, The Third Hospital of Nanchang, Nanchang, China; f Jiangxi Provincial People’s Hospital, Nanchang, China; g The First Affiliated Hospital of Jinan University, Guangzhou, China.

**Keywords:** immune trait, mediation, Mendelian randomization, puerperal sepsis, tea consumption

## Abstract

Puerperal sepsis is a special type of sepsis characterized by a poor prognosis. The relationship between tea consumption and puerperal sepsis remains controversial. This study aimed to investigate the causal relationship between tea consumption and the risk of puerperal sepsis, as well as to explore the mediating effects of immune traits. In this study, the Mendelian randomization (MR) analysis was used to explore the causal effect of tea intake on postnatal sepsis risk and mediators. Multiple methods were used used for verification. The 2-sample MR analysis showed a positive causal relationship between tea consumption and puerperal sepsis Inverse Variance Weighted (IVW) Inverse Variance Weighted: OR (odds ratio): 1.599 [1.034–2.472], *P* < .05). The conclusion was confirmed by a meta-analysis of 4 external validation datasets (IVW: OR: 1.303 [1.016–1.678], *P* < .05). Based on a 2-step MR analysis of 731 immune traits, we found that CD25 on IgD- CD38-B cell positively mediated the effect of tea on puerperal sepsis. Women who drink tea for a long time and in large quantities are potentially at high risk of postpartum sepsis and deserve attention.

## 1. Introduction

Puerperal sepsis is a fatal infectious disease with an extremely poor prognosis.^[1,2]^ The statistics showed that maternal sepsis accounts for 11% of maternal deaths, ranking as the third leading cause of maternal mortality worldwide after massive hemorrhage and hypertension.^[2,3]^ Therefore, the identification of high-risk pregnant women is critical for the prevention of puerperal sepsis.^[4]^

The World Health Organization has proposed the latest definition of sepsis (sepsis-3), which adds life-threatening organ dysfunction with the sequential organ failure assessment score ≥ 2.^[5]^ However, this definition is not applicable for maternal sepsis,^[6]^ and puerperal sepsis is distinct from the general sepsis. Tea is one of the most widely consumed beverages in the world.^[7]^ At the same time, it also constitutes a significant component of diets for women during postpartum recovery.^[8]^ The anti-inflammatory and antioxidant effects of tea are well known, which also suggests its protective role against sepsis in the general adult population.^[9,10]^ However, puerperal sepsis exhibits unique characteristics due to physiological changes during the perinatal period.^[6,11]^ Hachul et al found that rats consuming green tea extract during pregnancy and lactation had increased mesenteric IL-10/TNF-α ratio and IL-1β content, as well as elevated IL-1β content in retroperitoneal adipose tissue, alongside decreased catalase activity.^[12]^ Another study showed that maternal consumption of green tea extract induced a pro-inflammatory environment in the adipose tissue of adult offspring who received a control diet after weaning.^[13]^ Dey et al concluded that tea can increase the serum levels of pro-inflammatory cytokines while decreasing levels of anti-inflammatory cytokines.^[14]^ However, other studies have shown that tea can inhibit macrophage penetration and increase the expression of MAPK.^[15]^

The imbalanced immune response to infection is an important factor in the onset of sepsis.^[16]^ Postpartum, the body’s immune function to resist infection decreased due to the reconstruction of immune function, resulting in postpartum women being more susceptible to infections.^[17,18]^ Group A Streptococcus (GAS), the main pathogenic bacteria of puerperium sepsis, can lead to immune imbalance and puerperal sepsis by inhibiting/promoting immunity or interacting with immunomodulatory proteins.^[19,20]^ Therefore, immune factors play an important role in the development of puerperal sepsis.

Mendelian randomization (MR) analysis employs genetic variation linked to risk factors as Instrumental variables (IVs) to evaluate the presence of a causal effect, which can also effectively avoid bias and reverse causality that may result from confounding factors.^[21]^ Therefore, this study explored the causal relationship between tea consumption and puerperal sepsis risk through a 2-sample study and further explored the mediation role by pharmacological analysis and 2-step MR analysis.

## 2. Materials and methods

### 2.1. Data collection

We downloaded the tea intake-related dataset (ukb-b-6066, including 9851,867 single nucleotide polymorphisms (SNPs) and 447,485 individual samples) from the United Kingdom Biobank (https://www.ukbiobank.ac.uk/). The mean and standard deviation of tea consumption were 3.49631 and 2.84255 cups/day, respectively. The types of tea mainly include black tea and green tea. Data on tea consumption were derived from questionnaires. We used tea consumption (GCST009802, GCST90041729, GCST90096926, GCST90132983) from 4 GWAS (Genome-wide association study) Catalog databases (https://www.ebi.ac.uk/gwas/downloads/summary-statistics) for validation (Table S1, Supplemental Digital Content, https://links.lww.com/MD/Q173), and Meta-analysis was used to calculate the synthesis effect of validation datasets. Puerperal sepsis dataset (finngen_R11_O15_PUERP_SEPSIS, 248,744 individual samples) was obtained from the FinnGen (https://www.finngen.fi/en/access_results). The dataset of 731 immune traits was derived from the study of Orrù et al.^[22]^

### 2.2. Selection of instrumental variables

IVs in this study were selected by the following criteria: IVs were strongly correlated with the exposure: the criterion for selection of SNPs was *P* <5 × 10^−08^. If the number of SNPs did not meet the analysis requirements, the criterion was relaxed to “*P* <5 × 10^−06^” which is stricter than the standards used in previous studies.^[23,24]^ Exclusion of IVs with LD: the criterion was “linkage disequilibrium (LD) *r*^2^ <0.001 within a 10,000 kb distance.” IVs associated with confounding factors were excluded: LD trait database (https://ldlink.nci.nih.gov/?tab=ldtrait) helped us remove the confounding factors.^[25]^ Exclusion of weak IVs with *F* value < 10 (*F* = (N-2)*(2*((beta)^2)*eaf*(1-eaf))/(1-(2*((beta)^2)*eaf*(1-eaf))), beta: the effect size; eaf: the effect allele frequency).

### 2.3. Analysis methods

**Figure 1.**
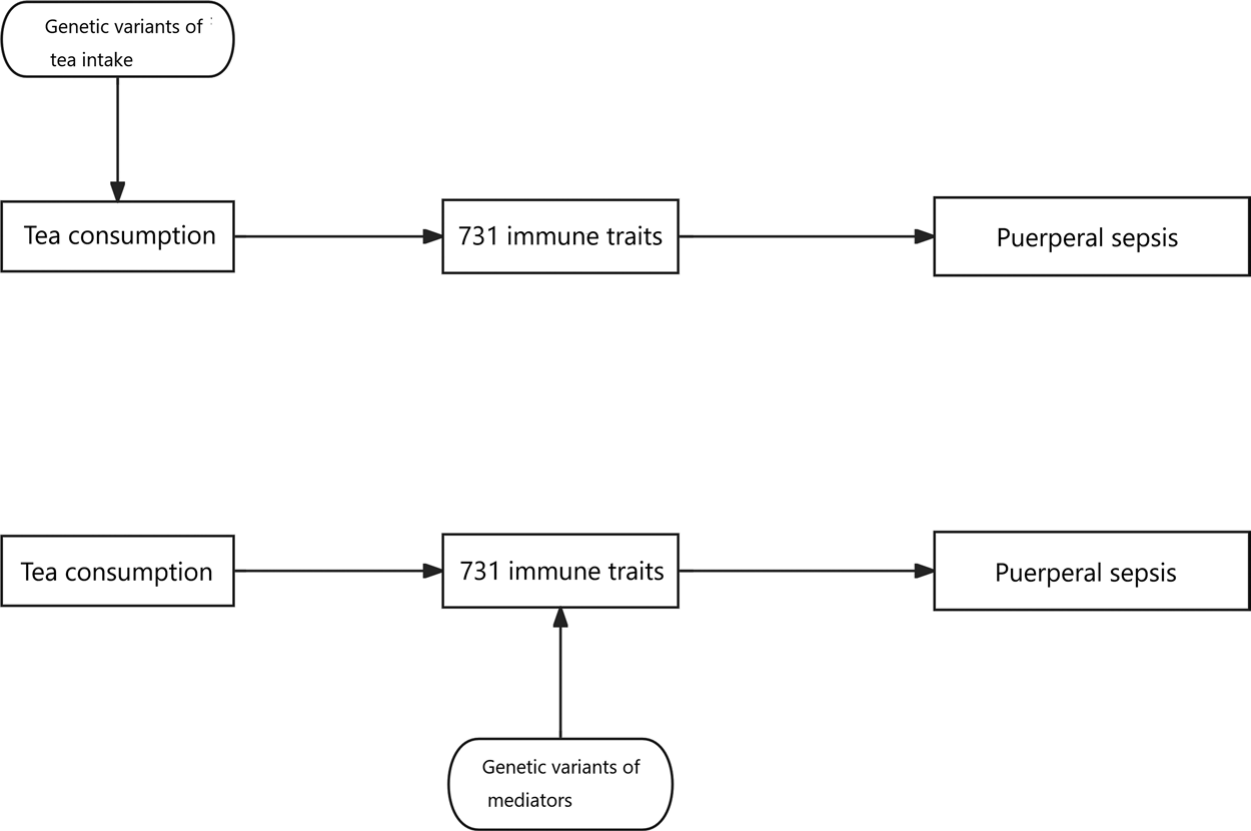
Flowchart of the study and MR analysis Step 1: genetic variant of tea intake is used as an instrument for the exposure of tea intake to estimate the causal impact of the exposure on mediators (identified by pharmacological analysis) of the association between the tea intake and the risk of puerperal sepsis; Step 2: genetic variant of mediators is used as an instrument for the mediators to establish the causal impact of the mediators on the risk of puerperal sepsis (causal effect of tea intake on the risk of puerperal sepsis (total effects) = beta0; causal effect of tea intake on the mediators = beta1; causal effect of the mediators on the risk of puerperal sepsis = beta2; mediating effects = beta1*beta2; direct effects = beta0-beta1*beta2). MR = Mendelian randomization.

In this study, the inverse variance weighted (IVW) was used as the primary method for MR analysis.^[26]^ Multiple methods (MR-Egger, weighted median, simple mode, weighted mode) were used to verify the consistency of the results. MR-Egger and IVW of Cochran *Q* were used to assess heterogeneity. The intercept of the MR-Egger regression was used to assess pleiotropy. The Leave-one-out method used to determine the causal relationship between exposure and outcome was not affected by a single SNP. All analyses were performed using the R language 4.3.1 software. *P* <.05 was considered to be statistically significant. The flowchart of our study and MR analysis was showed in Figure 1.

## 3. Results

### 3.1. The causal effect of tea consumption on puerperal sepsis

We obtained 4022 SNPs that were strongly related to tea consumption (*P* <5 × 10^−08^) in the ukb-b-6066 dataset. After removing LD, confounding variables (primarily with a diabetes-related SNP:^[27,28]^ rs57462170, rs2478875, rs9302428, rs9937354, rs2279844, rs12591786) and harmonizing data, the final 29 SNPs were retained to perform 2-sample MR analysis (effect of tea consumption on puerperal sepsis). *F*-statistics and the R² values of these SNPs were shown in Table S2, Supplemental Digital Content, https://links.lww.com/MD/Q173.

**Figure 2.**
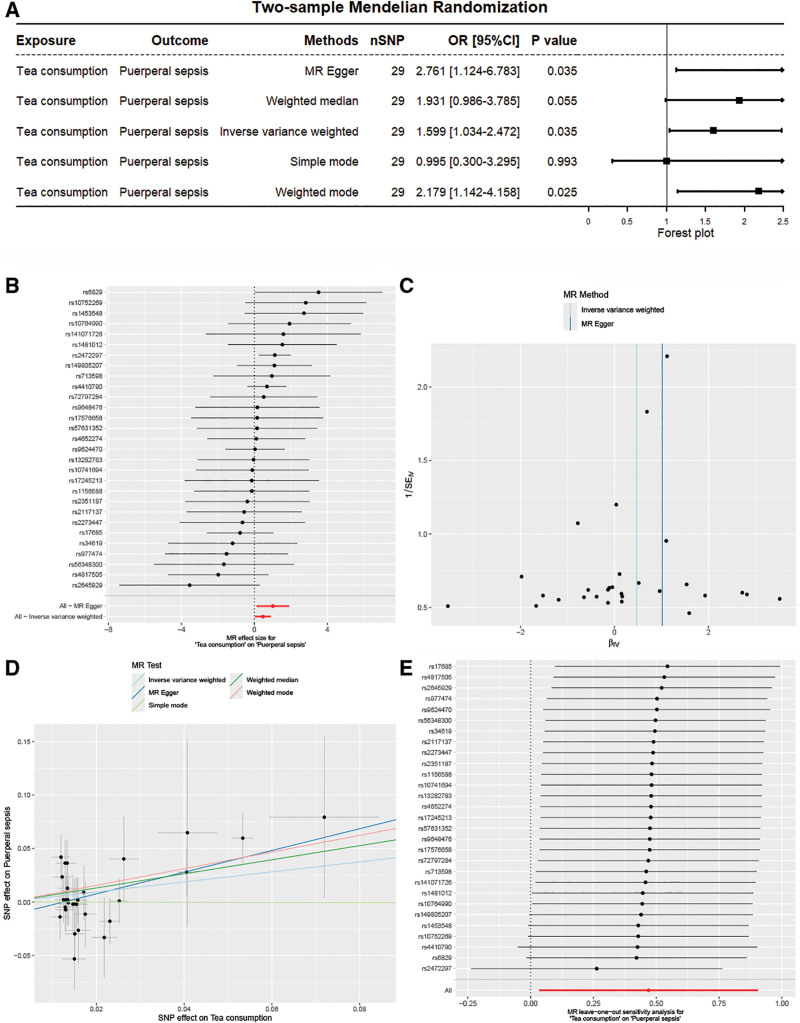
The causal effect of tea consumption on puerperal sepsis. (A) Genetically predicted increased tea consumption were associated with a increased risk of puerperal sepsis; (B) the effect size of each SNP for tea consumption on puerperal sepsis; (C) there was statistically no heterogeneity; (D) there was statistically no directional pleiotropy; (E) there was no apparent outlying SNPs. SNPs = single nucleotide polymorphisms

Two-sample MR analysis showed that genetically predicted tea consumption was positively associated with the risk of puerperal sepsis (IVW: OR: 1.599 [1.034–2.472], *P* < .05, Figure 2A). Moreover, the MR-Egger (OR: 2.761 [1.124–6.783], *P* < .05) and weighted mode (OR: 2.179 [1.142–4.158], *P* < .05) were consistent with the IVW results (Fig. 2A). The effect sizes of each IV on puerperal sepsis were shown in Figure 2B. Cochran *Q* statistics of MR-Egger and IVW (MR-Egger: Q = 23.321, *P* = .668; IVW: Q = 25.177, *P* = .618) did not show a significant heterogeneity (Fig. 2C). The intercept of the MR-Egger regression analysis did not show a significant pleiotropy, and the scatter plot was shown in Figure 2D. The Leave-One-Out method did not show any significantly outlying SNPs (Fig. 2E). Subsequently, we utilized 5 tea consumption datasets to verify the results. GCST90077558 still exhibited only 1 independent variable (IV) even after relaxing the *P*-value standard to 5 × 10^−06^, and thus it was not included in the verification.

### 3.2. The causal effect of tea consumption on puerperal sepsis in validation datasets and meta-analysis

**Figure 3.**
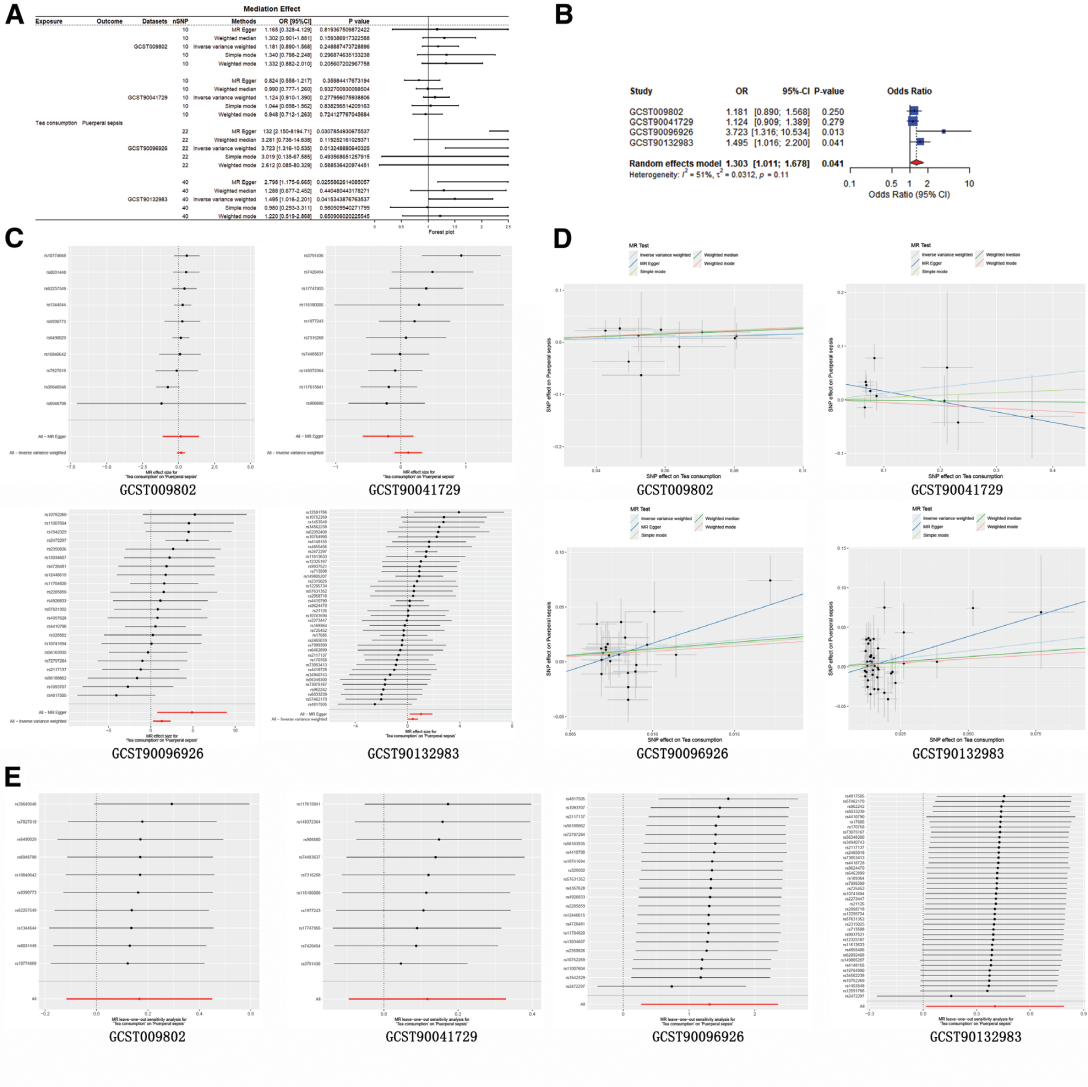
Validation of causal effect of tea consumption on puerperal sepsis. (A) Genetically predicted increased tea consumption were associated with a increased risk of puerperal sepsis in GCST90096926 and GCST90132983; (B) meta-analysis showed the total effect of tea consumption increased the risk of puerperal sepsis in validation datasets; (C) the effect size of each SNP for tea consumption on puerperal sepsis in validation datasets; (D) there was statistically no directional pleiotropy; (E) there was no apparent outlying SNPs in validation datasets. SNPs = single nucleotide polymorphisms

The result showed that tea consumption increased risk of puerperal sepsis in GCST90096926 and GCST90132983 datasets (Fig. 3A). *F*-statistics and the R² values of IVs of tea consumption were shown in Table S2, Supplemental Digital Content, https://links.lww.com/MD/Q173. The Meta-analysis showed that the synthesis effect of tea in the 4 data sets increased the risk of puerperal sepsis (Fig. 3B). Based on “*I*^2^ = 51%” and “Cochran *Q* = 6.12,” we selected the random effects model to describe the combined effect of the 4 datasets (OR: 1.303 [1.011, 1.678], *P* < .05). The effect sizes of each IV on puerperal sepsis in 4 datasets were shown in Figure 3C. There was no significant pleiotropy and outlying SNPs in 4 datasets (Fig. 3D and E).

### 3.3. The effect of immunological traits on puerperal sepsis

**Figure 4.**
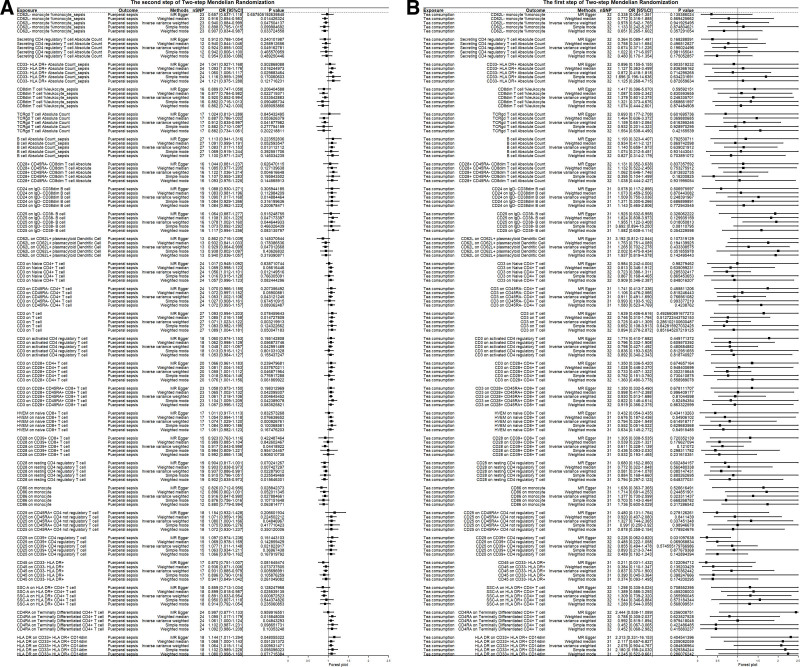
Two-step MR analysis for 26 immunological traits. (A) The MR analysis for the causal effect of tea consumption on 26 immunological traits; (B) the MR analysis for the causal effect of 26 immunological traits on puerperal sepsis. MR = Mendelian randomization.

In an exploratory 2-step MR analysis of 731 immune traits, we initially observed associations between 26 immunological traits and puerperal sepsis in the second step (*P* < .05, Fig. 4A). However, none of these immunological traits showed statistical significance after Bonferroni-adjusted significance level correction (*P* > .05/731). *F*-statistics and the R² values of IVs of these 26 immunological traits were shown in Table S3, Supplemental Digital Content, https://links.lww.com/MD/Q173.

### 3.4. The effect of tea consumption on immunological traits

In the first step of MR analysis, “CD25 on IgD- CD38- B cell” was nominally associated with tea consumption (*P* < .05; Fig. 4B). This trait did not survive after the test level adjustment (*P* > .05/26) but was prioritsized for further medation testing due to biological plausibility. The effects of individual SNPs on the 26 immune traits are shown in Figure S1, Supplemental Digital Content, https://links.lww.com/MD/Q174. Scatter plots (Fig. S2, Supplemental Digital Content, https://links.lww.com/MD/Q174) and Leave-One-Out analysis (Fig. S3, Supplemental Digital Content, https://links.lww.com/MD/Q174) indicated no significant pleiotropy or outlier SNPs for the associations of tea consumption with “CD25 on IgD- CD38- B cell” or this trait with puerperal sepsis.

## 4. Discussion

In our study, we found that the amount of tea consumption was positively associated with the risk of puerperal sepsis (IVW: OR: 1.599 [1.034–2.472], *P* < .05). Puerperal sepsis has characteristics that are distinct from those of general sepsis.^[6]^ Most studies believe that tea has anti-inflammation properties and may reduce the risk of sepsis.^[10,29–31]^ However, studies on puerperal sepsis have shown that tea has a pro-inflammatory effect,^[12–14]^ which is consistent with our findings.

Subsequently, we performed an exploratory 2-step MR analysis of 731 immunological traits. CD25 on IgD- CD38- B cell was identified as the mediation immunological trait for the causal effect of tea consumption on puerperal sepsis. Although there was no statistical significance after adjusting the test level with Bonferroni correction. Orrù et al defined the IgD-CD38-B cell as the late Bm5 memory cells.^[22]^ Lima et al found that the percentage of B cells at delivery and at postpartum was significantly lower than in the nonpregnant women and in the pregnant women during the 3rd trimester of pregnancy, and the absolute count of B cells at postpartum was significantly higher than at delivery.^[32]^ However, the absolute count of post-germinal memory/early Bm5 and the rate and the absolute count of resting memory/late Bm5 at postpartum were significantly higher than at delivery and at the 3rd trimester of pregnancy.^[32]^ The predominance of B cells in the postpartum shift to Bm5 implies a potential role for Bm5 in postpartum, which may potentially account for the different roles that tea plays in sepsis in general adults and postpartum women. The initial high inflammation and mediation of cytokines are important factors in sepsis pathogenesis.^[33]^ The study of Shin et al found that resting-state memory B cell population provoked a stronger immunoreactivity than naive B cells after antigenic stimulation.^[34]^ A variety of nutrients in tea can promote the proliferation and enhance the function of lymphocytes.^[35]^ In animal experiments, Huang et al found that epigallocatechin gallate can promote B cell proliferation.^[36]^ Wu et al found that tea affects the proliferation and activation of B cells, thereby increasing the risk of lung squamous cell carcinoma.^[37]^

The strengths of this study are that: the MR analysis was used in this study, which effectively controlled for confounding factors; the causal effect of tea consumption on the risk of puerperal sepsis were validated in this study by Meta-analysis, which enhanced the robustness of the results of MR analysis; multiple MR methods with different model assumptions were used to comprehensively evaluate the effects of outliers and pleiotropy, thereby verifying the consistency and reliability of the findings.

However, this study still has limitations: This study is based on MR randomization analysis and still needs to be validated in a large-scale population.

In conclusion, we found that genetically predicted tea consumption was positively associated with the risk of puerperal sepsis. The effect of tea consumption on puerperal sepsis risk was mediated by CD25 on IgD- CD38- B cell. However, further experimental and epidemiological studies are needed to verify this conclusion.

## Author contributions

**Data curation:** Wenyu Tong.

**Formal analysis:** Wenyu Tong.

**Funding acquisition:** Zherui Zhang.

**Investigation:** Wenyu Tong.

**Methodology:** Wenyu Tong, Yonghao Ouyang.

**Software:** Wenyu Tong, Yonghao Ouyang.

**Validation:** Wenyu Tong, Yonghao Ouyang.

**Visualization:** Yonghao Ouyang.

**Writing – original draft:** Wenyu Tong, Yonghao Ouyang.

**Writing – review & editing:** Wenyu Tong, Yonghao Ouyang, Beini Zhou, Wan Peng, Hong Liu, Yanru Xiang, Jinmiao Ye, Zherui Zhang.

## Supplementary Material


