# Supplementary material for: Causal effect of tea consumption on the increased risk of puerperal sepsis and the mediation effect of CD25 on IgD- CD38-B cell: A Mendelian randomization analysis
Source: Medicine (Baltimore). 2025 Oct 24;104(43):e44721. doi: 10.1097/MD.0000000000044721 (PMC12558255; doi:10.1097/MD.0000000000044721)
Supplement: Supplementary file 1 [file medi-104-e44721-s001.pdf]

Table S1 Information about the validation datasets

Datasets	Race	Sample size
GCST009802	Asian	152,653
GCST90041729	European	Cases: 50,994; controls: 13,007
GCST90096926	European	434,171
GCST90132983	European	448,060

Table S2 F-statistics and the R^2 values of instrumental variables (IVs) of tea consumption

Datasets	SNPs	Effect allele	Other allele	Beta exposure	Beta outcome	SE exposure	SE outcome	R2	F
ukb-b-6066	rs2645929	G	A	-0.0149842	0.0531722	0.0027166	0.0294194	6.82513455089048e-05	30.54340147060 8
	rs4817505	C	T	0.015068	-0.0298097	0.0021746	0.0212116	0.000108025833924873	48.3449467453922
	rs56348300	G	C	0.0158824	-0.0264935	0.00273191	0.0310609	7.59450087328981e-05	33.9866814616438
	rs977474	T	C	0.0217813	-0.0331722	0.00285559	0.0374547	0.000131523529692069	58.8622854127395
	rs34619	A	G	0.0117117	-0.0139325	0.0021375	0.0211814	6.72722847146639	30.10522902851

						e-05	05
rs17685	A	G	0.0230655	-0.0180136	0.00236195	0.0214927	0.000213337964965561	95.4854832553655
rs2273447	T	A	0.0174715	-0.0113595	0.00263421	0.0306618	9.9059859250797e-05	44.3319945182617
rs2117137	G	A	0.0129948	-0.00728599	0.0021557	0.0209722	8.13938929521704e-05	36.4253482008083
rs2351187	A	G	0.0129023	-0.00492496	0.0022823	0.022476	7.23194327488533e-05	32.3640572750187
rs1156588	G	A	-0.015454	0.00215922	0.00260325	0.0248871	7.92621725998689e-05	35.4712863127252
rs17245213	A	G	-0.01464	0.00203216	0.002609	0.027517	7.0705291	31.6416

			81		05	1	1419433e-05	530283598
rs10741694	C	T	0.0150037	-0.00168658	0.00219355	0.0236717	0.000105188862726868	47.075179642164
rs13282783	T	C	-0.0135837	0.000617177	0.00235432	0.0212824	7.53422240609293e-05	33.716904760496
rs9624470	A	G	0.0252071	0.000932638	0.00215485	0.0210142	0.000309554868826565	138.563434352863
rs4652274	C	T	-0.0157568	-0.00170419	0.0021744	0.0216563	0.000117822322173782	52.729698928659
rs57631352	G	A	-0.0131035	-0.00194297	0.00232117	0.0220596	7.16798791051371e-05	32.077826676342

rs17576658	A	G	-0.0134812	-0.00208708	0.00245655	0.0249395	6.76199283422747e-05	30.260814628502 8
rs9648476	A	G	0.0125013	0.00205974	0.00218542	0.0217392	7.34159912330547e-05	32.8548200741235
rs72797284	G	A	-0.0171147	-0.00881154	0.00238353	0.0256568	0.000115680690664 71	51.7711314209555
rs4410790	C	T	0.0405506	0.0279964	0.00219507	0.0221344	0.000765544987278398	342.830819958003
rs713598	G	C	0.0133969	0.0128757	0.00215659	0.0219007	8.63089189417113e-05	38.6251076660805
rs149805207	G	A	-0.0719337	-0.0792489	0.0125823	0.0754738	8.7604621666296e	39.20501345747

							-05	05
rs2472297	T	C	0.0533453	0.0597135	0.00240098	0.024151	0.00110060720529114	493.045663655252
rs1481012	G	A	-0.0262435	-0.0402859	0.00335608	0.0399323	0.000137218268664087	61.4112692646566
rs141071726	A	G	0.0407321	0.0648087	0.00681204	0.0881438	8.62714674034638e-05	38.6083458467544
rs10764990	A	G	-0.0121906	-0.0235114	0.00216898	0.0209596	7.08926097455478e-05	31.7254867893209
rs1453548	A	T	-0.0133414	-0.0362932	0.00224973	0.0222042	7.93130995921756e-05	35.494078890219 5
rs10752269	A	G	-0.01287	-0.0362927	0.0021197	0.021850	8.284094	37.0729

				27		5	7	34451938e-05	850567441
	rs6829	T	C	-0.0119163	-0.0419118	0.00216546	0.0213171	6.83733333615055e-05	30.5979964196159
GCST009802	rs6948798	T	G	0.0530322	-0.0629968	0.0113159	0.159389	0.00102715366947596	156.957254018591
	rs35640546	C	T	0.049418	-0.037043	0.0104537	0.0205434	0.001103890524128005	168.696214551371
	rs7827019	T	C	0.0640888	-0.00846736	0.0134323	0.0483051	0.000824584313014785	125.977499035517
	rs10840642	C	T	-0.0801924	-0.00798321	0.0166908	0.0590172	0.0011165836370042	170.638140527891

rs6490029	G	A	-0.0806833	-0.0123488	0.0092292	0.0244878	0.000888450767684623	135.743499554212
rs9390773	T	G	-0.0522687	-0.0133315	0.0107147	0.0330572	0.000907192061028575	138.609520764894
rs1344544	G	A	-0.0707159	-0.0198366	0.0141537	0.021983	0.00109699041141082	167.640583404831
rs62257549	A	G	-0.0587833	-0.0239979	0.011561	0.0256199	0.00117167127289 39	179.066598668121
rs8031448	C	T	0.0426791	0.0224616	0.00884887	0.0200941	0.000933981050931317	142.706426503917
rs10774668	A	G	-0.0468523	-0.0267213	0.00921073	0.0212693	0.00299255159011	458.1871414412

								415	06
GCST90041729	rs906680	G	A	0.0662811	-0.014276	0.0141083	0.0198677	0.00228512245842323	146.046853629025
	rs117615841	T	C	-0.232059	0.0423293	0.0456447	0.0515876	0.00203971886887865	130.33055107714
	rs149372364	A	G	-0.363185	0.0307889	0.0753982	0.0777987	0.00252406863714601	161.357036952608
	rs74483637	A	G	-0.207748	0.00213001	0.0434962	0.0475366	0.00221246340920996	141.39276807738
	rs7316268	C	T	0.0871159	0.00711485	0.0185594	0.0274766	0.00206249972340351	131.789172494365
	rs1977243	G	C	-0.07604	-0.0162608	0.014423	0.021310	0.002248	143.715

				75		9	7	73070593991	740192863
	rs116180006	G	T	-0.212476	-0.0599094	0.0463408	0.141148	0.00219621894021196	140.352341412068
	rs17747955	C	T	-0.0690229	-0.0271049	0.014067	0.0200738	0.00264229787600928	168.935143332017
	rs7420454	C	T	-0.0679668	-0.0331985	0.0148328	0.0217864	0.00220114780456648	140.668022013815
	rs3791436	T	C	-0.0833833	-0.0776449	0.0166692	0.0258223	0.00270776066769146	173.13186639416
GCST90096926	rs4817505	T	C	-0.00851247	0.0347249	0.001237	0.0200895	0.000147661	64.11934999
	rs1093707	T	G	0.008517	-0.0231138	0.001443	0.023338	7.1928E-0	31.2311

			36		15	6	5	6952
rs56188862	T	C	0.00687989	-0.0111556	0.00123844	0.0205219	7.23307E-05	31.40601575
rs2117137	A	G	-0.00850128	0.0101476	0.0012259	0.0198733	7.86781E-05	34.16225857
rs72797284	A	G	0.00895943	-0.00886498	0.00135539	0.0243192	7.36697E-05	31.98745422
rs56163935	T	C	-0.00901306	0.00287656	0.0015626	0.0214323	0.000110752	48.09041787
rs10741694	T	C	-0.00742105	-0.000600599	0.00124849	0.0224524	0.000103402	44.89847448
rs325502	G	A	-0.00689096	-0.00172567	0.0012184	0.0203148	8.13704E-05	35.33137824
rs4410790	T	C	-0.0114382	-0.0063807	0.00125129	0.0209867	7.74369E-05	33.62330276
rs4357628	A	C	0.00734106	0.00584959	0.00128144	0.0221672	7.50729E-05	32.59676202

rs57631352	A	G	0.00794761	0.00663905	0.00132017	0.0209027	7.64342E-05	33.18789712
rs4926833	G	A	-0.00697823	-0.00797226	0.00124868	0.0200069	7.10761E-05	30.86121695
rs2285859	A	G	-0.00714731	-0.0109835	0.00127537	0.0230483	9.15976E-05	39.77247988
rs11704820	A	G	0.00967218	0.0154229	0.0012395	0.0198797	7.71114E-05	33.48197985
rs12446615	A	G	0.0068673	0.0121357	0.00121187	0.0202958	0.000140228	60.89136057
rs4726481	G	T	-0.00714552	-0.0133366	0.00123489	0.0208409	0.000152302	66.13472432
rs13034607	C	T	-0.00725829	-0.0161879	0.00125174	0.0200429	7.39553E-05	32.11145402
rs2350826	T	G	-0.00832766	-0.0220788	0.00144555	0.0239959	7.66226E-05	33.26969997
rs2472297	C	T	-0.01719	-0.0742236	0.001362	0.022590	6.90878E-	29.9978

				71		75	7	05	3659
	rs1542329	G	A	0.00808051	0.0362445	0.00128129	0.0210154	8.34677E-05	36.24210182
	rs11007604	C	T	0.0101119	0.0456935	0.00177111	0.0274738	7.55839E-05	32.81867134
	rs10752269	G	A	0.00660431	0.0344612	0.00120582	0.0207148	0.000366657	159.2494906
GCST90132983	rs962242	T	C	-0.0153061	0.0283846	0.00250831	0.0232514	8.17557777997149e-05	36.6343253571484
	rs170768	C	T	0.0164374	-0.0133724	0.00214728	0.0205119	0.000128559429449023	57.6094870828604
	rs34562238	C	T	0.0137765	0.0334194	0.00245782	0.0217303	7.06057199045535e-05	31.6376914509755
	rs725452	A	G	0.014800	-0.0043674	0.002577	0.023592	7.264033	32.5494

			8	8	31	3	73553509e-05	486776965
rs57462170	G	A	-0.020364	0.0410699	0.00337004	0.0284077	8.06100001415942e-05	36.1208671465482
rs4855406	C	A	-0.0120786	-0.0197784	0.00218928	0.0202258	6.75310817212392e-05	30.259884896614 8
rs2117137	A	G	-0.0146544	0.0101476	0.00213156	0.0198733	0.000103508753650086	46.3827261611259
rs4148155	A	G	0.026191	0.0433403	0.00329408	0.0377228	0.000138377253198808	62.00961603
rs21126	G	A	-0.0127662	-0.000578481	0.00211847	0.0202995	7.99807304441549e-05	35.838872540551 2

rs2465018	G	A	-0.0214869	0.00860581	0.00250519	0.0255848	0.000163622699915396	73.3244572243534
rs149805207	A	G	0.0769244	0.0689749	0.012373	0.0717947	0.000101229016105473	45.3610623538975
rs4410790	T	C	-0.0384415	-0.0063807	0.0021744	0.0209867	0.000686978386458665	308.017763426016
rs73075167	A	T	0.0189998	-0.032914	0.00316937	0.0314511	8.15148489683698e-05	36.5263576395962
rs6462899	T	A	-0.012388	0.00683481	0.00216649	0.0206333	7.2087802343648e-05	32.301845111516
rs17685	G	A	-0.0219932	0.00664251	0.0023358	0.0203941	0.00019400060248	86.94038843449

							172	09
rs713598	C	G	-0.0140065	-0.0125619	0.00213445	0.0207428	9.42624556060737e-05	42.239028885316
rs2958718	C	T	0.0143767	0.00611578	0.00248081	0.0240978	7.41842661808826e-05	33.241319919399
rs56348300	C	G	-0.0167445	0.0285968	0.0027012 2	0.029481	8.4470063852217e-05	37.8506851192855
rs10752269	G	A	0.0123854	0.0344612	0.00209706	0.0207148	7.66839216817151e-05	34.3614795538729
rs4418728	G	T	0.0119409	-0.0121299	0.00210471	0.0197938	7.06020836721371e-05	31.6360619778652
rs10764990	G	A	0.013091	0.0292539	0.002145	0.019861	8.179312	36.6510

			3		79	3	18391879e-05	603896709
rs12295734	G	C	0.0127097	0.00636606	0.00229997	0.021805	6.70786710795804e-05	30.05715140034 7
rs10741694	T	C	-0.0154204	-0.000600599	0.00217077	0.0224524	0.000111103358985036	49.7862802430837
rs1453548	T	A	0.0131537	0.0361212	0.00222508	0.0210258	7.70824723150295e-05	34.5400808153507
rs73053413	C	T	0.0231021	-0.0205902	0.00283612	0.0356353	0.000145854882857107	65.360980321318
rs11613633	C	A	-0.0166777	-0.0232278	0.00291785	0.0257171	7.65587793272118e-05	34.3053999273346

rs7999399	C	T	-0.01181	0.00515479	0.00211072	0.0203758	6.88827353758934e-05	30.8655867530966
rs34940743	A	G	-0.0129371	0.0172299	0.00219485	0.0206938	7.59104685382516e-05	34.0148747973951
rs12591786	C	T	0.0190319	0.0748172	0.00291158	0.0334179	9.66075238205229e-05	43.2899560434355
rs2472297	C	T	-0.0517549	-0.0742236	0.00237716	0.0225907	0.0010406935777593	466.776854738651
rs12325187	C	G	0.0141191	0.0144166	0.00239297	0.0215089	7.70228422568137e-05	34.5133589729074
rs9937521	C	T	0.0137321	0.0126908	0.0021263	0.0199763	9.1956211536613e-	41.20550533083

							05	64
rs62092408	G	A	-0.015058	-0.0352727	0.002697	0.0300287	7.02344312244743e-05	31.4713091550739
rs57631352	A	G	0.0137484	0.00663905	0.00229623	0.0209027	7.89675384868573e-05	35.3848316123965
rs2315025	C	T	-0.0150955	-0.0109779	0.00222158	0.0230486	0.000101589250092638	45.5225008147275
rs6033239	G	T	0.0128219	-0.025552	0.00218202	0.0203976	7.60292705770798e-05	34.0681130900134
rs2273447	A	T	-0.0165222	0.00101561	0.00260399	0.0289823	8.86888119362342e-05	39.741256303331 6
rs4817505	T	C	-0.01387	0.0347249	0.002153	0.020089	9.176126	41.1181

			91		77	5	92742314e-05	438415427
rs9624470	G	A	-0.0261855	-0.00419355	0.00213225	0.0199075	0.00033397374515723	149.689600704217
rs169364	C	T	-0.0168492	0.00347462	0.00252786	0.023163	9.77684020804615e-05	43.8101979524161

Note: SNPs: single nucleotide polymorphisms; SE:standard error

Table S3 F-statistics of instrumental variables (IVs) of 26 immunological traits

Immune cells	SNPs	Effect allele	Other allele	Beta exposure	Beta outcome	SE exposure	SE outcome	F
CD62L- monocyte %monocyte	rs1816932	A	G	0.1467	-0.0654048	0.02962	0.0201991	24.5295804
CD62L- monocyte %monocyte	rs11249570	A	G	-0.1315	0.0417576	0.02634	0.0208228	33.5712813
CD62L- monocyte %monocyte	rs7150584	T	C	-0.1787	0.0343085	0.03646	0.0238236	24.0223658
CD62L- monocyte %monocyte	rs530377	C	T	0.1614	-0.0304471	0.03252	0.0221493	24.9692969
CD62L- monocyte %monocyte	rs11694457	C	T	0.5758	-0.10009	0.1297	0.0546152	19.7089446
CD62L- monocyte %monocyte	rs45487297	A	T	0.6623	-0.0913191	0.1201	0.118563	30.4104954
CD62L- monocyte %monocyte	rs2124027	G	C	-0.4256	0.0577757	0.09413	0.0257876	20.4431173

CD62L- monocyte %monocyte	rs9824107	A	C	-0.1976	0.0216082	0.04346	0.0265297	20.67256835
CD62L- monocyte %monocyte	rs2399610	G	A	-0.1315	0.0137577	0.02718	0.0198269	23.40736896
CD62L- monocyte %monocyte	rs17624563	G	C	-0.2904	0.0278604	0.05766	0.0326422	25.3655304
CD62L- monocyte %monocyte	rs76717104	T	C	0.4905	-0.0464046	0.109	0.0514222	20.25
CD62L- monocyte %monocyte	rs62236339	A	T	0.228	-0.019605	0.05043	0.0440342	20.44051142
CD62L- monocyte %monocyte	rs4987360	G	A	0.3359	-0.020097	0.02969	0.0226932	127.9969475
CD62L- monocyte %monocyte	rs117309559	G	A	0.5719	-0.0184245	0.1203	0.0474847	22.60002598
CD62L- monocyte %monocyte	rs141287837	C	T	0.3492	0.0153014	0.07853	0.0902893	19.77321439
CD62L- monocyte %monocyte	rs9859349	A	G	0.227	0.0134157	0.04151	0.028809	29.90516808
CD62L- monocyte %monocyte	rs55859756	C	G	0.2127	0.01428	0.04782	0.04860	19.7840789

					13		79	4
CD62L- monocyte %monocyte	rs9807014	T	C	-0.2057	-0.0163158	0.0397	0.0246419	26.84649354
CD62L- monocyte %monocyte	rs7139370	T	C	0.2947	0.0239961	0.0539	0.037529	29.89391128
CD62L- monocyte %monocyte	rs36141595	G	A	-0.1512	-0.0179973	0.03196	0.032413	22.38154389
CD62L- monocyte %monocyte	rs717824	T	C	-0.3108	-0.0529537	0.06924	0.0323375	20.14874042
CD62L- monocyte %monocyte	rs4656708	C	A	-0.3294	-0.0880083	0.06481	0.0878172	32.63287698
CD62L- monocyte %monocyte	rs4899389	A	G	-0.1913	-0.0602414	0.03943	0.0234139	23.53837004
Secreting CD4 regulatory T cell Absolute Count	rs17717645	C	T	0.3868	-0.126086	0.08192	0.0568788	22.29425907
Secreting CD4 regulatory T cell Absolute Count	rs75596315	T	C	0.2636	-0.0558164	0.05765	0.0321023	20.90702259

Secreting CD4 regulatory T cell Absolute Count	rs76202906	A	C	-0.3478	0.0491089	0.07473	0.108258	21.66053558
Secreting CD4 regulatory T cell Absolute Count	rs61839660	T	C	0.3436	-0.0327829	0.05734	0.0502919	35.90797657
Secreting CD4 regulatory T cell Absolute Count	rs149951966	C	T	0.2109	-0.0155449	0.04394	0.0252983	23.03737832
Secreting CD4 regulatory T cell Absolute Count	rs6942586	A	G	-0.1271	0.00876604	0.02687	0.0207186	22.37462515
Secreting CD4 regulatory T cell Absolute Count	rs7162344	A	G	-0.1209	0.00817217	0.02578	0.0217752	21.99311655
Secreting CD4 regulatory T cell Absolute Count	rs111992639	T	G	-0.7119	0.0390498	0.1247	0.074393	22.63150429
Secreting CD4 regulatory T cell Absolute Count	rs72915529	A	G	-0.5613	0.0103698	0.124	0.0463901	20.49022438
Secreting CD4 regulatory T cell Absolute Count	rs1041737	T	A	-0.1244	0.00137583	0.02796	0.0248454	19.79549776
Secreting CD4 regulatory T cell	rs3184504	C	T	-0.1297	-0.00370284	0.0282	0.0201526	21.1534756

Absolute Count								8
Secreting CD4 regulatory T cell Absolute Count	rs145600407	T	C	-0.5134	-0.118072	0.1045	0.149503	20.48109034
CD33- HLA DR+ Absolute Count	rs118076832	G	C	0.678	-0.0998366	0.1447	0.11759	21.95443806
CD33- HLA DR+ Absolute Count	rs139348198	T	C	-0.7561	0.0779802	0.16	0.196357	22.33153164
CD33- HLA DR+ Absolute Count	rs115139705	A	G	0.3782	-0.0356986	0.08437	0.0736667	20.09402558
CD33- HLA DR+ Absolute Count	rs876036	C	T	-0.2533	0.0233397	0.03729	0.0215378	28.61762179
CD33- HLA DR+ Absolute Count	rs6074118	T	A	-0.2049	0.0119298	0.04385	0.0317518	21.83457391
CD33- HLA DR+ Absolute Count	rs149034028	T	C	-0.4903	0.022425	0.104	0.0471633	22.22578495
CD33- HLA DR+ Absolute Count	rs3916927	A	G	-0.1801	0.00597903	0.03841	0.022846	21.98562476

CD33- HLA DR+ Absolute Count	rs6841247	T	A	0.2837	-0.00917106	0.06096	0.0377071	21.65851892
CD33- HLA DR+ Absolute Count	rs4955865	T	C	-0.1515	-0.00199254	0.0341	0.0204167	19.73860734
CD33- HLA DR+ Absolute Count	rs145935372	A	G	-0.6165	-0.0129449	0.137	0.119161	20.25
CD33- HLA DR+ Absolute Count	rs17883048	A	G	0.3306	0.0133307	0.07246	0.0661794	20.81656366
CD33- HLA DR+ Absolute Count	rs117803686	A	G	-0.6728	-0.0597442	0.1505	0.058052	19.98476132
CD33- HLA DR+ Absolute Count	rs34318562	A	G	-0.1942	-0.0176158	0.03571	0.0198259	29.57459124
CD33- HLA DR+ Absolute Count	rs185521042	C	A	0.7117	0.0659071	0.1571	0.154108	20.52303386
CD33- HLA DR+ Absolute Count	rs35720624	T	A	-0.3009	-0.0289433	0.0642	0.0958053	21.96718054
CD33- HLA DR+ Absolute	rs14336041	A	T	-0.6925	-0.0698544	0.156	0.179378	19.7056315

Count	1							7
CD33- HLA DR+ Absolute Count	rs116926801	T	C	-0.5549	-0.0666218	0.1175	0.052824	22.30250865
CD33- HLA DR+ Absolute Count	rs73023658	T	A	0.7644	0.0951555	0.1634	0.0806571	21.88453143
CD33- HLA DR+ Absolute Count	rs2705609	T	G	-0.2077	-0.0296408	0.04099	0.0253266	25.67539635
CD33- HLA DR+ Absolute Count	rs11695642	T	G	-0.1588	-0.0281411	0.03437	0.0200282	21.34724979
CD33- HLA DR+ Absolute Count	rs140563508	T	C	0.6759	0.130366	0.1402	0.113488	23.24175215
CD33- HLA DR+ Absolute Count	rs66733222	A	C	-0.1623	-0.0316024	0.03662	0.0304936	19.64266273
CD33- HLA DR+ Absolute Count	rs7207960	T	C	0.2569	0.0520833	0.05557	0.0274434	21.3721107
CD33- HLA DR+ Absolute Count	rs17382372	A	G	0.1961	0.0450768	0.04225	0.0278361	21.54278071

CD8dim T cell %leukocyte	rs9916257	T	G	0.1342	-0.0412453	0.0248	0.0203851	32.22610157
CD8dim T cell %leukocyte	rs112505246	C	T	0.2425	-0.0741003	0.04608	0.0677308	29.81293989
CD8dim T cell %leukocyte	rs11676464	A	G	-0.1769	0.0453661	0.03963	0.0355637	23.40256882
CD8dim T cell %leukocyte	rs73558811	G	A	-0.5587	0.107474	0.1206	0.0876676	21.27640237
CD8dim T cell %leukocyte	rs8022863	G	A	0.1228	-0.0220385	0.0274	0.0208835	20.02339828
CD8dim T cell %leukocyte	rs139249541	G	A	0.5228	-0.0921802	0.07237	0.0827563	31.80806706
CD8dim T cell %leukocyte	rs79086781	T	C	0.471	-0.0776429	0.06515	0.0656076	57.46618106
CD8dim T cell %leukocyte	rs112072415	A	T	0.275	-0.0364045	0.05986	0.0344554	20.16327387
CD8dim T cell %leukocyte	rs62287820	G	A	0.4434	-0.0575767	0.09405	0.0435147	20.5358606

								4
CD8dim T cell %leukocyte	rs143576769	T	C	-0.2522	0.0224006	0.05621	0.0920631	19.85746653
CD8dim T cell %leukocyte	rs17700958	A	T	-0.3157	0.0227744	0.07015	0.0400409	21.2305335
CD8dim T cell %leukocyte	rs111748563	G	A	0.2703	0.0247531	0.057	0.0503852	26.83763636
CD8dim T cell %leukocyte	rs60409696	T	C	0.3554	0.0362332	0.07361	0.0912593	20.52217394
CD8dim T cell %leukocyte	rs12871857	T	C	-0.2405	-0.0334855	0.05208	0.0274146	22.88458729
CD8dim T cell %leukocyte	rs2937553	G	A	-0.1298	-0.0228185	0.02605	0.025128	23.58199165
CD8dim T cell %leukocyte	rs34740679	A	G	0.1531	0.0408274	0.03448	0.0324364	19.71586642
TCRgd T cell Absolute Count	rs9912354	T	C	0.109	-0.0473372	0.0245	0.0207709	19.79341941
TCRgd T cell Absolute Count	rs558986	G	A	-0.1539	0.0552795	0.03343	0.0276882	21.1935879

								5
TCRgd T cell Absolute Count	rs117810965	A	G	0.2763	-0.0781076	0.06238	0.0718649	19.61873626
TCRgd T cell Absolute Count	rs35702634	A	G	0.2848	-0.0795935	0.05856	0.0537331	23.65254263
TCRgd T cell Absolute Count	rs7192379	C	T	-0.1323	0.025832	0.02955	0.0231372	20.04493803
TCRgd T cell Absolute Count	rs8079417	G	T	-0.2623	0.0489878	0.0573	0.0480918	20.95498418
TCRgd T cell Absolute Count	rs3802329	A	C	0.1504	-0.0192441	0.03066	0.0200183	24.06308867
TCRgd T cell Absolute Count	rs10850589	T	C	-0.1129	0.0144127	0.02418	0.020493	21.80094357
TCRgd T cell Absolute Count	rs73353488	A	G	-0.2867	0.0325015	0.0603	0.0318135	22.60584584
TCRgd T cell Absolute Count	rs149000203	A	G	-0.3381	0.02169	0.07544	0.0632694	20.08570048

TCRgd T cell Absolute Count	rs9974788	A	G	0.4148	-0.00264225	0.09106	0.133189	20.75019601
TCRgd T cell Absolute Count	rs7194802	A	C	-0.1303	-0.00444885	0.02779	0.0253194	21.98425424
TCRgd T cell Absolute Count	rs139311612	C	T	-0.3512	-0.0159675	0.07076	0.0691	24.63391088
TCRgd T cell Absolute Count	rs11897347	C	T	0.154	0.024613	0.03447	0.0278646	19.95992361
TCRgd T cell Absolute Count	rs947107	C	T	-0.1373	-0.0237688	0.02868	0.0260876	22.91832008
TCRgd T cell Absolute Count	rs148395664	A	G	0.3857	0.0884947	0.08384	0.0627871	21.16395057
TCRgd T cell Absolute Count	rs142067182	T	C	0.2644	0.0930576	0.05643	0.164033	21.95345109
B cell Absolute Count	rs35606919	G	T	0.1779	-0.0421248	0.03926	0.057518	20.53294792
B cell Absolute Count	rs58114937	G	A	0.1852	-0.0246515	0.03578	0.0468025	21.9839567

								7
B cell Absolute Count	rs6750281	G	A	0.1112	-0.0146297	0.02488	0.021183	19.97601348
B cell Absolute Count	rs11666499	G	A	0.1913	-0.0122101	0.0422	0.0238457	20.5496788
B cell Absolute Count	rs10052651	T	A	-0.1161	0.007236	0.02565	0.0236924	20.48753463
B cell Absolute Count	rs2033489	T	C	0.1073	-0.00546858	0.02416	0.0207656	19.72448097
B cell Absolute Count	rs7834992	G	A	0.1209	-0.00488791	0.02608	0.0231094	21.49005008
B cell Absolute Count	rs182527768	A	G	-0.3328	0.010542	0.06652	0.0608084	25.03007519
B cell Absolute Count	rs11627066	T	C	-0.1147	0.00144886	0.02444	0.0198989	22.38979334
B cell Absolute Count	rs4933299	T	G	-0.1282	0.000299043	0.02869	0.0204657	19.9670982
B cell Absolute Count	rs117693242	G	A	-0.4542	-0.0134175	0.09774	0.0789823	21.5948201

B cell Absolute Count	rs17113368	T	C	0.1875	0.00680747	0.04172	0.0302006	20.19825936
B cell Absolute Count	rs72941136	C	G	0.1802	0.00893261	0.03974	0.027327	20.58594615
B cell Absolute Count	rs1897558	C	A	-0.1305	-0.00821042	0.02801	0.0224961	21.70675006
B cell Absolute Count	rs75270114	G	T	0.2916	0.0212325	0.06479	0.0639579	20.25625145
B cell Absolute Count	rs12865434	G	A	0.1537	0.0155859	0.02404	0.0244764	40.87698055
B cell Absolute Count	rs13173982	A	G	0.1243	0.0138408	0.02807	0.0329689	19.60908965
B cell Absolute Count	rs73520180	C	T	0.4493	0.0537269	0.08473	0.0358995	28.11890629
B cell Absolute Count	rs1966302	C	T	-0.1213	-0.0153446	0.02419	0.0199401	25.14489723
B cell Absolute Count	rs7500855	T	C	-0.1258	-0.0160624	0.02435	0.0197738	26.6909081

								7
B cell Absolute Count	rs2679580	G	A	-0.1187	-0.0186932	0.02392	0.0206549	19.82178924
B cell Absolute Count	rs2395904	G	T	-0.1269	-0.022734	0.02725	0.022442	23.01552038
B cell Absolute Count	rs9883798	A	C	0.1124	0.0229529	0.02525	0.0218878	19.81572003
B cell Absolute Count	rs7210666	C	T	-0.129	-0.027364	0.02915	0.0219482	19.58404067
B cell Absolute Count	rs62061834	C	T	0.2608	0.0678345	0.05507	0.052086	22.42771461
B cell Absolute Count	rs5009526	G	A	-0.1516	-0.0456106	0.03391	0.0307106	19.98677958
B cell Absolute Count	rs1057686	G	A	-0.1735	-0.0613539	0.0364	0.0265818	22.71936511
CD28+ CD45RA- CD8dim T cell Absolute Count	rs138678985	A	G	0.2897	-0.0732812	0.06184	0.0856681	20.44491122

CD28+ CD45RA- CD8dim T cell Absolute Count	rs1450949	A	G	0.1131	-0.0234584	0.02512	0.0239939	20.27150252
CD28+ CD45RA- CD8dim T cell Absolute Count	rs35222020	G	A	0.3244	-0.0134646	0.07107	0.0353586	20.83478677
CD28+ CD45RA- CD8dim T cell Absolute Count	rs2585739	A	G	0.2989	0.00149612	0.06454	0.0715716	21.44835099
CD28+ CD45RA- CD8dim T cell Absolute Count	rs565801	G	T	-0.1068	-0.000755191	0.02285	0.0198435	21.84590781
CD28+ CD45RA- CD8dim T cell Absolute Count	rs13231184	G	C	-0.4138	-0.0293591	0.09073	0.0321322	20.8007575
CD28+ CD45RA- CD8dim T cell Absolute Count	rs35035328	C	T	-0.2541	-0.0212739	0.05644	0.0296053	20.26913989
CD28+ CD45RA- CD8dim T cell Absolute Count	rs4129441	C	T	-0.1161	-0.0130451	0.02528	0.0219314	21.09163758
CD28+ CD45RA- CD8dim T cell Absolute Count	rs138728019	T	C	-0.4332	-0.0578712	0.09651	0.0590709	20.14801496
CD28+ CD45RA- CD8dim T cell	rs61269136	T	C	0.2626	0.0481875	0.058	0.0351923	20.4990368

Absolute Count								6
CD28+ CD45RA- CD8dim T cell Absolute Count	rs687264	C	T	-0.2594	-0.0568319	0.05546	0.0311112	23.01081277
CD28+ CD45RA- CD8dim T cell Absolute Count	rs28527279	A	G	-0.1214	-0.0309145	0.02643	0.0239675	21.09808546
CD28+ CD45RA- CD8dim T cell Absolute Count	rs2670003	C	T	-0.1279	-0.0447099	0.0272	0.0207895	22.11074016
CD28+ CD45RA- CD8dim T cell Absolute Count	rs12235775	C	T	0.1255	0.0457547	0.02537	0.0202845	25.89636258
CD28+ CD45RA- CD8dim T cell Absolute Count	rs116884829	G	A	0.1788	0.118147	0.03398	0.0666357	20.48078321
CD28+ CD45RA- CD8dim T cell Absolute Count	rs116499679	T	C	0.235	0.568732	0.05229	0.325721	20.19753833
CD24 on IgD- CD38dim B cell	rs7606151	C	A	0.1171	-0.0395116	0.02513	0.0209045	21.713449
CD24 on IgD- CD38dim B cell	rs61969021	C	T	0.1267	-0.0219835	0.02653	0.0261391	22.80755495
CD24 on IgD- CD38dim B cell	rs35629713	A	C	0.3684	-0.0428809	0.07227	0.051384	25.9850246

								8
CD24 on IgD- CD38dim B cell	rs77636548	C	T	-0.3035	0.0199131	0.06696	0.110691	26.73586745
CD24 on IgD- CD38dim B cell	rs142131620	T	C	-0.1626	0.00243062	0.03562	0.0339547	20.83786654
CD24 on IgD- CD38dim B cell	rs147397792	G	T	0.2105	0.00508943	0.04421	0.0902349	22.67060815
CD24 on IgD- CD38dim B cell	rs12642193	G	A	-0.168	-0.00781055	0.0337	0.021487	20.57743892
CD24 on IgD- CD38dim B cell	rs7756279	A	C	-0.1139	-0.0053289	0.02562	0.0199642	19.76465315
CD24 on IgD- CD38dim B cell	rs6561591	C	T	-0.1184	-0.00714072	0.02497	0.019828	22.48362432
CD24 on IgD- CD38dim B cell	rs72948545	G	A	-0.7439	-0.0519473	0.05193	0.0584345	283.8096926
CD24 on IgD- CD38dim B cell	rs61820496	A	G	-0.2216	-0.0209013	0.04504	0.0605499	24.20709912

CD24 on IgD- CD38dim B cell	rs2236073	A	G	0.1758	0.0195586	0.02511	0.0198078	52.12164057
CD24 on IgD- CD38dim B cell	rs117417471	G	A	-0.2819	-0.0340392	0.061	0.0406724	21.35651975
CD24 on IgD- CD38dim B cell	rs7532008	C	A	0.1141	0.0156813	0.02516	0.0223994	20.5660087
CD24 on IgD- CD38dim B cell	rs783418	C	G	-0.2057	-0.0324903	0.03306	0.0387023	21.15724147
CD24 on IgD- CD38dim B cell	rs59949425	C	A	0.3167	0.0658322	0.0704	0.04545	20.23721793
CD24 on IgD- CD38dim B cell	rs10777178	C	T	-0.131	-0.0331553	0.0289	0.0214986	20.54692832
CD24 on IgD- CD38dim B cell	rs1363793	A	G	-0.1362	-0.0379204	0.02875	0.0252383	22.44287637
CD24 on IgD- CD38dim B cell	rs12930821	C	G	-0.1566	-0.0585977	0.02914	0.0209632	21.04666637
CD25 on IgD- CD38- B cell	rs117534066	T	C	0.4471	-0.0915345	0.1005	0.0724345	19.7914319

CD25 on IgD- CD38- B cell	rs115945675	C	T	0.2558	-0.0434438	0.05745	0.0488	19.8253569
CD25 on IgD- CD38- B cell	rs144182927	T	G	-0.4156	0.0436949	0.08772	0.0952005	19.5997046 8
CD25 on IgD- CD38- B cell	rs11036291	G	A	-0.1424	0.0124106	0.03012	0.0238197	22.3516734 3
CD25 on IgD- CD38- B cell	rs1570675	C	T	0.1132	-0.00365009	0.02483	0.0203423	25.8539444 6
CD25 on IgD- CD38- B cell	rs11245200	T	C	0.1611	-0.00274196	0.03564	0.029022	20.6354190 6
CD25 on IgD- CD38- B cell	rs2180045	C	T	0.11	0.00251762	0.02464	0.020012	19.9298469 4
CD25 on IgD- CD38- B cell	rs4073186	A	G	-0.1155	-0.00467776	0.025	0.0199966	21.0099734 5
CD25 on IgD- CD38- B cell	rs80243585	G	A	0.2012	0.0121741	0.04222	0.024464	22.7101234 4
CD25 on IgD- CD38- B cell	rs12342539	T	C	-0.1657	-0.0166815	0.02956	0.0221409	31.4221688

								1
CD25 on IgD- CD38- B cell	rs74911560	G	C	-0.3809	-0.0415584	0.08077	0.0513788	22.23933411
CD25 on IgD- CD38- B cell	rs16957211	G	C	0.1436	0.0158749	0.03202	0.0234207	20.11250776
CD25 on IgD- CD38- B cell	rs12722531	A	G	0.4479	0.0698741	0.04714	0.176182	90.27821604
CD25 on IgD- CD38- B cell	rs706779	C	T	-0.293	-0.0463273	0.02511	0.0204224	136.1575773
CD25 on IgD- CD38- B cell	rs11694298	A	G	0.1206	0.0493761	0.02623	0.0207827	21.13966177
CD25 on IgD- CD38- B cell	rs73374860	C	T	-0.3425	-0.171111	0.07563	0.128801	20.50845532
CD62L on CD62L+ plasmacytoid Dendritic Cell	rs150038452	C	G	0.4551	-0.147992	0.08329	0.0623652	29.85574734
CD62L on CD62L+ plasmacytoid Dendritic Cell	rs62236308	T	G	-0.1699	0.0439901	0.03364	0.0295943	25.50790457

CD62L on CD62L+ plasmacytoid Dendritic Cell	rs343813	T	A	-0.2089	0.0377251	0.03169	0.0225893	43.4542643 1
CD62L on CD62L+ plasmacytoid Dendritic Cell	rs149154085	G	T	-0.2147	0.0373178	0.04734	0.0695676	20.5687349 4
CD62L on CD62L+ plasmacytoid Dendritic Cell	rs613872	T	G	-0.1931	0.0186344	0.04309	0.0270047	20.0822125 5
CD62L on CD62L+ plasmacytoid Dendritic Cell	rs11773763	T	C	-0.1856	0.0143984	0.03003	0.0212762	38.1984094 3
CD62L on CD62L+ plasmacytoid Dendritic Cell	rs4987358	T	G	0.3259	-0.0223412	0.02935	0.024124	123.297
CD62L on CD62L+ plasmacytoid Dendritic Cell	rs148535663	T	C	-0.1954	0.00787833	0.04378	0.050068	19.9203793 4
CD62L on CD62L+ plasmacytoid Dendritic Cell	rs4731265	A	G	0.1345	-0.00403193	0.02728	0.0205705	24.3083699 2
CD62L on CD62L+ plasmacytoid Dendritic Cell	rs12864970	A	C	0.1188	0.00571258	0.02579	0.0198761	21.2192581 8
CD62L on CD62L+	rs14089342	A	G	-0.3623	-0.0183975	0.0776	0.035388	21.7978282

plasmacytoid Dendritic Cell	2							1
CD62L on CD62L+ plasmacytoid Dendritic Cell	rs12040885	A	G	-0.1178	-0.0177309	0.02609	0.0198034	20.38648832
CD62L on CD62L+ plasmacytoid Dendritic Cell	rs111528892	A	G	0.4028	0.116277	0.08171	0.149838	24.30124382
CD3 on Naive CD4+ T cell	rs146330992	T	C	0.2517	-0.0811116	0.04997	0.0878251	25.37159278
CD3 on Naive CD4+ T cell	rs113731203	C	T	-0.4916	0.121298	0.1065	0.0955891	21.30710926
CD3 on Naive CD4+ T cell	rs74744824	G	A	0.3526	-0.0351629	0.07893	0.0579934	23.64156213
CD3 on Naive CD4+ T cell	rs61814872	T	C	0.3354	-0.0246149	0.04422	0.0332832	41.39619529
CD3 on Naive CD4+ T cell	rs118065562	A	G	-0.6044	0.0307008	0.1307	0.0802487	21.47173509
CD3 on Naive CD4+ T cell	rs9318267	G	T	0.1354	-0.00632576	0.03002	0.0212067	20.34304468

CD3 on Naive CD4+ T cell	rs139257983	G	A	-0.332	0.00782669	0.07384	0.102307	20.21588654
CD3 on Naive CD4+ T cell	rs28607988	C	A	0.2334	-0.00521016	0.03629	0.0337223	41.36449885
CD3 on Naive CD4+ T cell	rs112171230	T	C	0.3132	-0.00189379	0.06122	0.032113	20.43535372
CD3 on Naive CD4+ T cell	rs11715223	A	G	0.1539	0.000345813	0.03327	0.022016	20.08870183
CD3 on Naive CD4+ T cell	rs4925475	C	T	0.1214	0.00113	0.02737	0.0201793	19.67377855
CD3 on Naive CD4+ T cell	rs2213429	T	C	-0.1619	-0.00787424	0.03478	0.0266703	21.66878409
CD3 on Naive CD4+ T cell	rs2949661	T	C	0.6864	0.0376611	0.02442	0.0227097	482.977285
CD3 on Naive CD4+ T cell	rs4657649	C	T	0.5489	0.0421792	0.06086	0.0315759	60.72864044
CD3 on Naive CD4+ T cell	rs2413113	C	T	0.13	0.0121966	0.02724	0.0199554	24.00964351

CD3 on Naive CD4+ T cell	rs34064330	G	T	-0.1285	-0.0151554	0.02897	0.0198618	19.67475192
CD3 on Naive CD4+ T cell	rs113208949	T	C	0.6287	0.0742182	0.1285	0.109971	23.93760329
CD3 on Naive CD4+ T cell	rs28535434	C	T	0.1895	0.0246464	0.04111	0.022465	21.24826505
CD3 on Naive CD4+ T cell	rs12502302	T	C	0.1301	0.0190063	0.02774	0.0221967	21.99589712
CD3 on Naive CD4+ T cell	rs4396110	A	G	-0.1303	-0.022489	0.0274	0.0223978	19.90681331
CD3 on Naive CD4+ T cell	rs7502332	A	G	-0.1403	-0.0246781	0.0284	0.0204804	24.40499157
CD3 on Naive CD4+ T cell	rs146993166	T	C	0.2723	0.0613003	0.06124	0.0742038	19.77083076
CD3 on Naive CD4+ T cell	rs7530127	C	T	-0.1373	-0.0420808	0.0278	0.021586	24.39222866
CD3 on Naive CD4+ T cell	rs62232208	G	A	-0.1325	-0.0430066	0.02751	0.022549	27.2179163

								9
CD3 on CD45RA- CD4+ T cell	rs9925166	T	C	0.1959	-0.048278	0.03814	0.0514618	24.06117759
CD3 on CD45RA- CD4+ T cell	rs12884372	G	T	0.235	-0.0316516	0.05221	0.0818308	19.78494699
CD3 on CD45RA- CD4+ T cell	rs12497487	T	C	-0.143	0.014285	0.03198	0.0212403	26.79924796
CD3 on CD45RA- CD4+ T cell	rs61814872	T	C	0.3354	-0.0246149	0.04422	0.0332832	41.39619529
CD3 on CD45RA- CD4+ T cell	rs59302791	T	C	0.4066	-0.0306229	0.0812	0.0402532	20.25274567
CD3 on CD45RA- CD4+ T cell	rs7536105	C	T	0.1302	-0.0096495	0.02735	0.0198338	22.66247339
CD3 on CD45RA- CD4+ T cell	rs188669685	G	C	0.2305	-0.0148769	0.0503	0.168504	22.82255725
CD3 on CD45RA- CD4+ T cell	rs57319220	A	G	0.1904	-0.00879657	0.03623	0.0336061	30.48054424

CD3 on CD45RA- CD4+ T cell	rs11015146	C	T	-0.1451	0.00290503	0.0326	0.0269982	19.81069103
CD3 on CD45RA- CD4+ T cell	rs11715223	A	G	0.1539	0.000345813	0.03327	0.022016	20.08870183
CD3 on CD45RA- CD4+ T cell	rs1317631	T	C	-0.1828	-0.000596818	0.03573	0.0280611	22.71221385
CD3 on CD45RA- CD4+ T cell	rs7595804	C	T	-0.2491	-0.00335539	0.05165	0.0337312	19.77017756
CD3 on CD45RA- CD4+ T cell	rs3809841	T	G	-0.1309	-0.00421241	0.0286	0.0200645	20.25935659
CD3 on CD45RA- CD4+ T cell	rs72940579	T	C	-0.1452	-0.00818898	0.03122	0.0219608	19.84117142
CD3 on CD45RA- CD4+ T cell	rs148932595	A	C	-0.6105	-0.0394654	0.1289	0.116454	22.43187116
CD3 on CD45RA- CD4+ T cell	rs2949661	T	C	0.6864	0.0376611	0.02442	0.0227097	482.977285
CD3 on CD45RA- CD4+ T cell	rs72732798	A	G	0.235	0.019483	0.05212	0.102981	20.32950997

CD3 on CD45RA- CD4+ T cell	rs12141563	G	A	-0.1858	-0.0168198	0.03779	0.0244612	24.17339427
CD3 on CD45RA- CD4+ T cell	rs4657649	C	T	0.5489	0.0421792	0.06086	0.0315759	60.72864044
CD3 on CD45RA- CD4+ T cell	rs28649534	T	C	0.2373	0.0335035	0.04656	0.045297	24.49175405
CD3 on CD45RA- CD4+ T cell	rs1077544	T	A	-0.1562	-0.0260536	0.03369	0.0209712	21.49611798
CD3 on CD45RA- CD4+ T cell	rs4396110	A	G	-0.1303	-0.022489	0.0274	0.0223978	19.90681331
CD3 on CD45RA- CD4+ T cell	rs62232208	G	A	-0.1325	-0.0430066	0.02751	0.022549	27.21791639
CD3 on CD45RA- CD4+ T cell	rs138909872	T	C	-0.4512	-0.157854	0.09903	0.0872287	20.75891372
CD3 on T cell	rs62029957	A	G	0.1923	-0.0679611	0.04324	0.0684807	26.12863155
CD3 on T cell	rs18580520	A	C	-0.5943	0.145453	0.1329	0.172893	19.9968458

	8							4
CD3 on T cell	rs61618798	A	C	-0.1743	0.0151861	0.03461	0.046754	25.36247171
CD3 on T cell	rs2520957	T	C	-0.1416	0.0101685	0.02854	0.0237104	24.61606155
CD3 on T cell	rs55799444	T	C	-0.1333	0.00663711	0.02896	0.0204197	21.1866935
CD3 on T cell	rs2122146	C	T	-0.153	0.00721049	0.03421	0.0227994	20.00215154
CD3 on T cell	rs75466245	C	T	0.2635	-0.00807344	0.05844	0.0305797	20.33016131
CD3 on T cell	rs10996702	A	G	0.1468	-0.00315329	0.03117	0.0218755	22.18086643
CD3 on T cell	rs35750035	C	A	0.1927	-0.00268905	0.04288	0.0262965	20.19546587
CD3 on T cell	rs6684311	G	C	-0.2191	0.000528327	0.0385	0.0228393	36.11680819
CD3 on T cell	rs11612873	A	C	0.4577	0.00687138	0.09839	0.0641648	21.6401319

	1							1
CD3 on T cell	rs148114970	C	A	0.2756	0.0112076	0.06064	0.0305219	20.65570589
CD3 on T cell	rs17614424	T	C	0.4615	0.0245246	0.09698	0.0388344	22.64535072
CD3 on T cell	rs59684757	G	T	0.1421	0.00822661	0.02938	0.0214466	23.3929274
CD3 on T cell	rs148932595	A	C	-0.6105	-0.0394654	0.1289	0.116454	22.43187116
CD3 on T cell	rs6479155	C	T	-0.1306	-0.0100877	0.02685	0.0199484	21.42657474
CD3 on T cell	rs1617988	C	T	0.428	0.0368085	0.02617	0.0228205	267.4730886
CD3 on T cell	rs41289614	T	C	0.1602	0.0142691	0.03447	0.0332412	21.59943827
CD3 on T cell	rs1529714	A	G	-0.1386	-0.013912	0.02984	0.0227966	21.07518116
CD3 on T cell	rs4456952	T	C	-0.3884	-0.0415391	0.08427	0.0554727	21.2428339

								6
CD3 on T cell	rs4657649	C	T	0.5489	0.0421792	0.06086	0.0315759	60.72864044
CD3 on T cell	rs114672530	A	G	-0.52	-0.0619842	0.104	0.0766099	25
CD3 on T cell	rs28636970	G	A	0.5589	0.0734488	0.1093	0.0436732	26.14736295
CD3 on T cell	rs12729972	T	C	-0.1371	-0.0231308	0.02745	0.0223801	24.94538505
CD3 on T cell	rs12591149	A	G	0.1257	0.0215254	0.02752	0.019872	20.86285291
CD3 on T cell	rs75169391	T	C	-0.344	-0.0614843	0.07014	0.0370253	24.0538923
CD3 on T cell	rs1502523	A	G	0.1262	0.0373671	0.02696	0.0197812	21.91184434
CD3 on activated CD4 regulatory T cell	rs8024202	A	G	-0.1286	0.0129393	0.0277	0.0220083	21.29129199
CD3 on activated CD4 regulatory	rs59302791	T	C	0.4066	-0.0306229	0.0812	0.0402532	20.2527456

T cell								7
CD3 on activated CD4 regulatory T cell	rs148303369	C	T	0.3289	-0.0225774	0.06852	0.0750628	23.04056042
CD3 on activated CD4 regulatory T cell	rs142664794	T	C	0.3527	-0.00924036	0.07928	0.0340841	19.79172447
CD3 on activated CD4 regulatory T cell	rs4767280	T	C	-0.124	0.00232555	0.02782	0.0200029	19.86685486
CD3 on activated CD4 regulatory T cell	rs72758215	C	T	-0.1412	0.000181356	0.0319	0.0243837	19.59241753
CD3 on activated CD4 regulatory T cell	rs12138291	A	G	-0.2802	-0.000889248	0.0384	0.0228932	53.24438477
CD3 on activated CD4 regulatory T cell	rs78694250	A	G	-0.4581	-0.00385823	0.0996	0.0411574	21.15445819
CD3 on activated CD4 regulatory T cell	rs552999	A	C	-0.1644	-0.00160632	0.03044	0.0207441	29.16851573
CD3 on activated CD4 regulatory T cell	rs59684757	G	T	0.1421	0.00822661	0.02938	0.0214466	23.3929274

CD3 on activated CD4 regulatory T cell	rs2949661	T	C	0.6864	0.0376611	0.02442	0.0227097	482.977285
CD3 on activated CD4 regulatory T cell	rs4728966	G	A	0.2231	0.014708	0.04962	0.0353276	20.2155527 5
CD3 on activated CD4 regulatory T cell	rs114672530	A	G	-0.52	-0.0619842	0.104	0.0766099	25
CD3 on activated CD4 regulatory T cell	rs78043002	G	T	0.3228	0.033036	0.0698	0.0379546	21.3873120 9
CD3 on activated CD4 regulatory T cell	rs35055340	T	C	-0.4927	-0.0640371	0.07664	0.0444526	41.3289272 2
CD3 on activated CD4 regulatory T cell	rs138786273	A	G	-0.2927	-0.051968	0.06601	0.063548	19.6619224 7
CD3 on activated CD4 regulatory T cell	rs4396110	A	G	-0.1303	-0.022489	0.0274	0.0223978	19.9068133 1
CD3 on activated CD4 regulatory T cell	rs12401936	G	A	0.1738	0.0398421	0.03767	0.0254244	21.2866982 5
CD3 on CD28+ CD4+ T cell	rs18580520	A	C	-0.5943	0.145453	0.1329	0.172893	19.9968458

	8							4
CD3 on CD28+ CD4+ T cell	rs1922987	C	T	0.1317	-0.0191402	0.02851	0.0207723	21.33914995
CD3 on CD28+ CD4+ T cell	rs75149813	C	T	0.309	-0.0402101	0.06652	0.0439081	21.57806404
CD3 on CD28+ CD4+ T cell	rs61618798	A	C	-0.1743	0.0151861	0.03461	0.046754	25.36247171
CD3 on CD28+ CD4+ T cell	rs2520957	T	C	-0.1416	0.0101685	0.02854	0.0237104	24.61606155
CD3 on CD28+ CD4+ T cell	rs61814886	A	G	0.2351	-0.0113169	0.04363	0.0318647	51.61151585
CD3 on CD28+ CD4+ T cell	rs62001340	A	G	0.3979	-0.0125159	0.08629	0.117397	21.26311756
CD3 on CD28+ CD4+ T cell	rs73048160	T	C	0.3676	-0.00770612	0.07432	0.0907843	24.4646836
CD3 on CD28+ CD4+ T cell	rs11787185	C	A	-0.1223	0.00115437	0.02741	0.0198864	19.90833055
CD3 on CD28+ CD4+ T cell	rs11542399	T	C	0.2839	-0.00035069	0.06342	0.0514934	20.0391044

	8							8
CD3 on CD28+ CD4+ T cell	rs78457979	G	T	-0.3464	-0.0100122	0.07548	0.0294356	21.06163023
CD3 on CD28+ CD4+ T cell	rs62237850	A	G	0.1888	0.0108805	0.04252	0.0318663	19.71593911
CD3 on CD28+ CD4+ T cell	rs78042544	G	A	0.4923	0.0324968	0.09143	0.0385749	20.98769804
CD3 on CD28+ CD4+ T cell	rs41289614	T	C	0.1602	0.0142691	0.03447	0.0332412	21.59943827
CD3 on CD28+ CD4+ T cell	rs2988277	T	C	0.4749	0.0377535	0.02583	0.0227752	338.030219
CD3 on CD28+ CD4+ T cell	rs4657649	C	T	0.5489	0.0421792	0.06086	0.0315759	60.72864044
CD3 on CD28+ CD4+ T cell	rs4396110	A	G	-0.1303	-0.022489	0.0274	0.0223978	19.90681331
CD3 on CD28+ CD4+ T cell	rs113348527	A	G	0.4416	0.0778093	0.09394	0.091302	22.098206
CD3 on CD28+ CD4+ T cell	rs14413718	T	C	0.2524	0.0519035	0.04859	0.113854	26.9826688

	5							2
CD3 on CD28+ CD4+ T cell	rs2728811	G	T	0.1223	0.0440928	0.02723	0.0201597	20.17240279
CD3 on CD28+ CD45RA+ CD8+ T cell	rs148876959	T	C	-0.2674	0.0926816	0.05986	0.065335	19.95489196
CD3 on CD28+ CD45RA+ CD8+ T cell	rs62028293	C	T	0.253	-0.0819792	0.05243	0.0694608	20.55819461
CD3 on CD28+ CD45RA+ CD8+ T cell	rs111780443	T	C	-0.2919	0.0406104	0.06527	0.0940572	30.71664503
CD3 on CD28+ CD45RA+ CD8+ T cell	rs7519927	A	G	-0.1899	0.0123674	0.02764	0.0203089	29.48377643
CD3 on CD28+ CD45RA+ CD8+ T cell	rs116266532	C	T	0.3159	-0.0190381	0.06429	0.119836	24.14417667
CD3 on CD28+ CD45RA+ CD8+ T cell	rs4646296	G	C	-0.3431	0.00705003	0.07418	0.0277028	21.0477267
CD3 on CD28+ CD45RA+ CD8+ T cell	rs2713986	T	C	-0.2046	0.00329211	0.03907	0.0258538	27.42359814

CD3 on CD28+ CD45RA+ CD8+ T cell	rs12138291	A	G	-0.2802	-0.000889248	0.0384	0.0228932	53.24438477
CD3 on CD28+ CD45RA+ CD8+ T cell	rs7998384	A	G	0.3703	0.00382714	0.07919	0.0282981	21.86586881
CD3 on CD28+ CD45RA+ CD8+ T cell	rs4656440	G	C	-0.3384	-0.00408097	0.06295	0.037452	25.42659998
CD3 on CD28+ CD45RA+ CD8+ T cell	rs2995089	A	G	0.6021	0.037729	0.02516	0.0227755	595.7529298
CD3 on CD28+ CD45RA+ CD8+ T cell	rs6479155	C	T	-0.1306	-0.0100877	0.02685	0.0199484	21.42657474
CD3 on CD28+ CD45RA+ CD8+ T cell	rs12921174	C	T	-0.1808	-0.0150942	0.0337	0.0200002	32.38024561
CD3 on CD28+ CD45RA+ CD8+ T cell	rs4657649	C	T	0.5489	0.0421792	0.06086	0.0315759	60.72864044
CD3 on CD28+ CD45RA+ CD8+ T cell	rs78043002	G	T	0.3228	0.033036	0.0698	0.0379546	21.38731209
CD3 on CD28+ CD45RA+ CD8+	rs79844317	T	C	-0.3872	-0.0467645	0.08711	0.138522	19.8455617

T cell								1
CD3 on CD28+ CD45RA+ CD8+ T cell	rs73526900	C	T	0.4501	0.0661376	0.09557	0.0782879	22.18067987
CD3 on CD28+ CD45RA+ CD8+ T cell	rs1442353	T	G	-0.131	-0.0209911	0.02946	0.021673	19.7732067
CD3 on CD28+ CD45RA+ CD8+ T cell	rs2235394	G	T	-0.1308	-0.0211768	0.02732	0.0202743	22.92208391
CD3 on CD28+ CD45RA+ CD8+ T cell	rs79617518	T	C	0.6198	0.104538	0.1291	0.0470997	23.04892418
CD3 on CD28+ CD45RA+ CD8+ T cell	rs12729972	T	C	-0.1371	-0.0231308	0.02745	0.0223801	24.94538505
CD3 on CD28+ CD45RA+ CD8+ T cell	rs12990220	A	G	-0.1751	-0.0366424	0.03614	0.024667	23.47448081
CD3 on CD28+ CD45RA+ CD8+ T cell	rs73099996	A	G	-0.2602	-0.0891499	0.05667	0.0540229	21.08182283
HVEM on naive CD8+ T cell	rs112421400	T	C	-0.2471	0.0324341	0.05362	0.0544048	31.97652426

HVEM on naive CD8+ T cell	rs17105684	A	G	-0.4536	0.0107616	0.09893	0.10871	21.02277652
HVEM on naive CD8+ T cell	rs2523589	T	G	0.2311	0.00223479	0.04267	0.0197565	29.33287322
HVEM on naive CD8+ T cell	rs1353750	C	T	-0.3769	-0.00478603	0.08324	0.0236471	20.50161775
HVEM on naive CD8+ T cell	rs28422844	G	A	0.5661	0.0150848	0.124	0.0464164	20.84217027
HVEM on naive CD8+ T cell	rs73851787	G	A	0.5851	0.0297411	0.1309	0.0299389	19.97932921
HVEM on naive CD8+ T cell	rs12476985	C	T	0.8901	0.0473839	0.1957	0.0528507	20.68692552
HVEM on naive CD8+ T cell	rs561768	G	A	-0.1931	-0.0131286	0.04187	0.0240332	21.26956588
HVEM on naive CD8+ T cell	rs28904611	G	A	0.4581	0.0379529	0.09597	0.0618357	22.78502943
HVEM on naive CD8+ T cell	rs11815170	T	C	0.4464	0.038346	0.09669	0.048924	21.3149958

	2							
HVEM on naive CD8+ T cell	rs2234161	T	C	0.2305	0.0202085	0.03896	0.0198049	35.00289482
HVEM on naive CD8+ T cell	rs17614093	G	C	0.1964	0.0196053	0.04379	0.0201844	20.11560396
HVEM on naive CD8+ T cell	rs2844594	A	G	-0.276	-0.0303232	0.04325	0.0288625	40.72357914
HVEM on naive CD8+ T cell	rs4898532	C	T	-0.1882	-0.0235535	0.04006	0.0198192	22.07076305
HVEM on naive CD8+ T cell	rs2964678	T	C	-0.29	-0.041595	0.05954	0.024031	23.72347667
HVEM on naive CD8+ T cell	rs10090960	G	A	-0.1865	-0.0323879	0.04018	0.0204686	21.54456885
HVEM on naive CD8+ T cell	rs11981290	A	G	-0.2026	-0.0495317	0.04387	0.0222353	21.32767987
CD28 on CD39+ CD8+ T cell	rs2028827	A	G	-0.2498	0.0967401	0.05432	0.0382527	21.14780735

CD28 on CD39+ CD8+ T cell	rs902972	T	C	-0.2934	0.099958	0.06379	0.0544485	21.15509669
CD28 on CD39+ CD8+ T cell	rs3116493	T	G	0.1976	-0.0619629	0.02744	0.0255987	131.5284283
CD28 on CD39+ CD8+ T cell	rs11070382	C	A	-0.24	0.0714106	0.05412	0.0303764	19.6655867
CD28 on CD39+ CD8+ T cell	rs10847448	C	G	-0.1275	0.021284	0.02786	0.0200915	20.94392844
CD28 on CD39+ CD8+ T cell	rs72871333	T	C	0.1359	-0.0202821	0.02951	0.0203685	21.20803808
CD28 on CD39+ CD8+ T cell	rs77213229	C	T	-0.6487	0.0341652	0.1258	0.0861623	26.59050061
CD28 on CD39+ CD8+ T cell	rs17490604	C	T	-0.1427	0.00672822	0.03169	0.0224194	20.27698911
CD28 on CD39+ CD8+ T cell	rs148345105	C	T	0.4419	-0.00521576	0.09833	0.0669858	20.19649121
CD28 on CD39+ CD8+ T cell	rs10237725	T	C	0.1318	-0.000528321	0.02811	0.0205956	21.98412219

CD28 on CD39+ CD8+ T cell	rs79635002	G	A	0.4585	0.00177896	0.09178	0.0357917	24.95643651
CD28 on CD39+ CD8+ T cell	rs77594357	A	T	-0.1443	-0.00156063	0.03049	0.0273263	30.30183915
CD28 on CD39+ CD8+ T cell	rs624763	T	C	-0.132	-0.012342	0.0294	0.0236746	20.15826739
CD28 on CD39+ CD8+ T cell	rs143273680	G	A	0.4873	0.0563072	0.09956	0.076568	23.95648225
CD28 on CD39+ CD8+ T cell	rs7177745	A	C	-0.1385	-0.0243648	0.02922	0.0210568	22.46669159
CD28 on resting CD4 regulatory T cell	rs72692959	G	A	0.4971	-0.184491	0.1068	0.0938942	21.66431795
CD28 on resting CD4 regulatory T cell	rs112900587	A	G	-0.245	0.0742107	0.05172	0.0571538	22.4396013
CD28 on resting CD4 regulatory T cell	rs643902	T	C	0.1937	-0.0371865	0.04207	0.0373555	21.1989435
CD28 on resting CD4 regulatory	rs14492575	A	G	0.5643	-0.0786495	0.1145	0.05951	24.2889716

T cell	0							1
CD28 on resting CD4 regulatory T cell	rs72923091	A	G	0.1434	-0.0190041	0.02945	0.0216992	23.70978984
CD28 on resting CD4 regulatory T cell	rs62184016	C	T	-0.5184	0.0648065	0.03354	0.0300125	146.6743236
CD28 on resting CD4 regulatory T cell	rs2583662	G	A	-0.1355	0.0166858	0.02829	0.0202504	22.94101852
CD28 on resting CD4 regulatory T cell	rs72928038	A	G	0.5273	-0.0450105	0.03289	0.0311162	28.41120525
CD28 on resting CD4 regulatory T cell	rs6872582	T	C	-0.1337	0.00899851	0.02867	0.0217376	21.74740431
CD28 on resting CD4 regulatory T cell	rs12662612	C	A	0.1433	-0.0065668	0.02939	0.0216145	23.77350461
CD28 on resting CD4 regulatory T cell	rs79223025	G	A	0.192	-0.00335377	0.0411	0.02732	21.82321914
CD28 on resting CD4 regulatory T cell	rs7665484	T	C	0.1309	0.00409768	0.02845	0.0199271	21.16970234

CD28 on resting CD4 regulatory T cell	rs4877468	T	C	0.1366	0.00471776	0.02838	0.020565	23.16736369
CD28 on resting CD4 regulatory T cell	rs35141413	G	C	-0.2066	-0.0138266	0.04634	0.0319133	19.87689646
CD28 on resting CD4 regulatory T cell	rs1834937	A	G	0.1241	0.0134487	0.02781	0.0234172	19.91322428
CD28 on resting CD4 regulatory T cell	rs75440037	T	C	-0.3832	-0.0549893	0.0864	0.0982553	19.67086763
CD28 on resting CD4 regulatory T cell	rs139910716	A	G	0.3236	0.0672666	0.07039	0.0513722	21.13465172
CD28 on resting CD4 regulatory T cell	rs138480662	C	G	-0.6131	-0.141632	0.1328	0.0838072	21.31407463
CD28 on resting CD4 regulatory T cell	rs34562294	C	T	0.2776	0.122107	0.06218	0.220728	19.93138546
CD86 on monocyte	rs72663570	T	C	0.1555	-0.0319897	0.03506	0.0246399	19.67147672
CD86 on monocyte	rs61300354	A	G	0.4396	-0.084008	0.09917	0.0961803	19.6496464

								7
CD86 on monocyte	rs6559641	A	C	0.5595	-0.102256	0.1131	0.0465847	24.47231037
CD86 on monocyte	rs150649461	C	G	-0.4543	0.0767499	0.07237	0.0609657	20.96804354
CD86 on monocyte	rs150328798	C	T	0.541	-0.064098	0.1177	0.0596214	21.12718351
CD86 on monocyte	rs56283543	G	A	0.2202	-0.0195408	0.04384	0.02469	25.2286225
CD86 on monocyte	rs10169719	T	C	0.6001	-0.028464	0.1324	0.0687195	20.54335085
CD86 on monocyte	rs7565243	G	A	-0.1388	0.00394551	0.02986	0.0199665	21.60724147
CD86 on monocyte	rs12974366	A	G	0.1425	0.0166145	0.03134	0.0307972	20.6743443
CD86 on monocyte	rs7857137	G	A	-0.1263	-0.0232455	0.02794	0.022735	20.43402393
CD86 on monocyte	rs138552617	T	C	-0.2153	-0.048267	0.04558	0.0589942	22.3120471

CD86 on monocyte	rs73592377	T	C	-0.2179	-0.06416	0.04813	0.0622439	20.49664301
CD25 on CD45RA+ CD4 not regulatory T cell	rs34644690	G	A	-0.2825	0.077549	0.06255	0.0491422	20.39775054
CD25 on CD45RA+ CD4 not regulatory T cell	rs494642	T	C	-0.1675	0.0364243	0.03766	0.0403725	19.78194506
CD25 on CD45RA+ CD4 not regulatory T cell	rs116411520	A	G	-0.3549	0.0657533	0.0748	0.0964162	22.51172646
CD25 on CD45RA+ CD4 not regulatory T cell	rs9838853	C	G	-0.1242	0.0124488	0.02748	0.0262463	20.42724204
CD25 on CD45RA+ CD4 not regulatory T cell	rs1699228	A	C	0.1183	-0.0114919	0.02664	0.0219219	19.71973381
CD25 on CD45RA+ CD4 not regulatory T cell	rs55751874	A	C	0.1166	-0.0058032	0.02528	0.0219362	21.27369662
CD25 on CD45RA+ CD4 not regulatory T cell	rs936056	G	A	-0.1251	-0.0046908	0.02543	0.0225712	24.20036404
CD25 on CD45RA+ CD4 not	rs10905719	A	G	0.2281	0.00981176	0.02982	0.0206047	23.5849820

regulatory T cell								1
CD25 on CD45RA+ CD4 not regulatory T cell	rs34779614	T	C	-0.2226	-0.0151807	0.04896	0.0280307	20.67128628
CD25 on CD45RA+ CD4 not regulatory T cell	rs2279434	T	C	0.2554	0.0219737	0.05665	0.0349116	20.32553368
CD25 on CD45RA+ CD4 not regulatory T cell	rs9921355	T	C	0.1116	0.0118239	0.02452	0.0207032	20.71512019
CD25 on CD45RA+ CD4 not regulatory T cell	rs1385469	G	A	0.1547	0.0170057	0.03457	0.0256266	20.02543083
CD25 on CD45RA+ CD4 not regulatory T cell	rs62026958	C	G	0.1218	0.0135892	0.02548	0.0204679	22.85050115
CD25 on CD45RA+ CD4 not regulatory T cell	rs16917989	C	A	-0.1633	-0.0273529	0.03619	0.0310718	20.36081713
CD25 on CD45RA+ CD4 not regulatory T cell	rs75432369	G	A	-0.3926	-0.0975978	0.08754	0.0504657	20.11349334
CD25 on CD45RA+ CD4 not regulatory T cell	rs9298702	C	T	-0.1211	-0.0417531	0.0268	0.0225983	20.41825852

CD25 on CD45RA+ CD4 not regulatory T cell	rs73112886	A	T	-0.2336	-0.0914708	0.04984	0.0444793	21.96795395
CD25 on CD45RA+ CD4 not regulatory T cell	rs74458586	G	A	-0.5528	-0.225007	0.1157	0.130507	22.82807816
CD25 on CD39+ CD4 regulatory T cell	rs11968423	T	C	0.1834	-0.0249503	0.03686	0.0311129	24.75642906
CD25 on CD39+ CD4 regulatory T cell	rs113703605	A	G	0.2428	-0.0242631	0.05419	0.0461166	20.07516219
CD25 on CD39+ CD4 regulatory T cell	rs7477011	A	G	-0.1133	0.00428791	0.0254	0.0201864	19.89721929
CD25 on CD39+ CD4 regulatory T cell	rs7078614	T	G	0.182	-0.00236424	0.02618	0.0205977	27.16788398
CD25 on CD39+ CD4 regulatory T cell	rs17516976	A	T	-0.1194	0.000766114	0.02573	0.0233111	21.5342168
CD25 on CD39+ CD4 regulatory T cell	rs71526028	A	G	0.2385	0.0111702	0.04928	0.0261255	23.42261438
CD25 on CD39+ CD4 regulatory	rs209493	C	G	-0.5296	-0.036574	0.1168	0.0261703	20.5593920

T cell								1
CD25 on CD39+ CD4 regulatory T cell	rs182193538	A	C	0.4366	0.0382256	0.09258	0.093899	22.23991432
CD25 on CD39+ CD4 regulatory T cell	rs6434720	G	A	0.3841	0.043208	0.08113	0.040763	22.41432488
CD25 on CD39+ CD4 regulatory T cell	rs205775	T	C	-0.1192	-0.0185168	0.02679	0.0200259	19.79735103
CD25 on CD39+ CD4 regulatory T cell	rs1840456	C	A	0.2302	0.0418072	0.04935	0.0708828	21.75886935
CD25 on CD39+ CD4 regulatory T cell	rs61960319	A	G	0.5226	0.116836	0.1155	0.0710189	20.47268679
CD25 on CD39+ CD4 regulatory T cell	rs75122374	G	C	0.3127	0.0858025	0.06895	0.0935151	20.56777068
CD25 on CD39+ CD4 regulatory T cell	rs1506123	G	T	0.1233	0.0474062	0.02588	0.024267	22.69852356
CD25 on CD39+ CD4 regulatory T cell	rs11861637	T	C	0.3202	0.198898	0.06753	0.129804	22.48273573

CD45 on CD33- HLA DR+	rs17552904	T	G	-0.2523	0.0480829	0.04091	0.0244243	38.03431001
CD45 on CD33- HLA DR+	rs143150964	A	G	-0.6499	0.1230211	0.1405	0.0531234	21.39638606
CD45 on CD33- HLA DR+	rs3124947	C	G	-0.2363	0.0363266	0.04925	0.0213553	23.02051174
CD45 on CD33- HLA DR+	rs12523072	T	C	0.2734	-0.0376198	0.05833	0.0433286	21.96914056
CD45 on CD33- HLA DR+	rs4980766	T	C	0.2168	-0.0276753	0.04667	0.0276711	21.57957832
CD45 on CD33- HLA DR+	rs10971439	C	T	0.1762	-0.0221858	0.03942	0.0274108	19.9792218
CD45 on CD33- HLA DR+	rs1326284	C	A	0.2087	-0.0218413	0.03919	0.0276288	28.35922584
CD45 on CD33- HLA DR+	rs10735302	T	C	-0.1815	0.0129583	0.03652	0.0213453	24.69970242
CD45 on CD33- HLA DR+	rs33978622	C	G	0.3661	-0.0236076	0.04284	0.0206239	149.6838732

CD45 on CD33- HLA DR+	rs11222974	T	C	-0.7691	0.0269144	0.1667	0.0867979	21.2860179
CD45 on CD33- HLA DR+	rs117317282	T	A	0.2989	-0.00552849	0.06723	0.0323574	19.76631024
CD45 on CD33- HLA DR+	rs1678025	G	A	0.1667	-0.00257562	0.03644	0.0207093	20.92736086
CD45 on CD33- HLA DR+	rs149228799	T	C	-0.3721	-0.00689052	0.08366	0.0766893	19.78261138
CD45 on CD33- HLA DR+	rs34358471	C	T	-0.2401	-0.00610088	0.05182	0.0284062	21.46789699
CD45 on CD33- HLA DR+	rs11232456	C	A	0.1768	0.00756109	0.03944	0.0215891	20.09512485
CD45 on CD33- HLA DR+	rs71466481	C	G	-0.2245	-0.0115483	0.04663	0.0237401	23.17938237
CD45 on CD33- HLA DR+	rs9892517	G	A	0.2156	0.0339697	0.04742	0.0336777	20.67161528
SSC-A on HLA DR+ CD4+ T cell	rs151184302	A	G	0.3142	-0.141372	0.06758	0.0899515	21.61603259

SSC-A on HLA DR+ CD4+ T cell	rs3104376	C	T	-0.16	0.0483611	0.03351	0.0233339	35.94422389
SSC-A on HLA DR+ CD4+ T cell	rs353628	C	G	-0.181	0.0539625	0.04075	0.0218879	19.72885694
SSC-A on HLA DR+ CD4+ T cell	rs7805250	G	A	0.2848	-0.0717419	0.06354	0.0277751	20.09025962
SSC-A on HLA DR+ CD4+ T cell	rs13159652	T	C	-0.1566	0.0336272	0.03208	0.0267209	23.82949266
SSC-A on HLA DR+ CD4+ T cell	rs4924305	A	G	0.1205	-0.0161224	0.02701	0.0206776	22.93685987
SSC-A on HLA DR+ CD4+ T cell	rs72679223	C	T	0.387	-0.0425423	0.08251	0.0337623	21.99929461
SSC-A on HLA DR+ CD4+ T cell	rs6498113	G	T	-0.155	0.0164813	0.03288	0.0245568	22.22285121
SSC-A on HLA DR+ CD4+ T cell	rs78800399	A	G	0.5031	-0.0518823	0.113	0.0628405	19.82219516
SSC-A on HLA DR+ CD4+ T	rs1155750	G	A	-0.2309	0.0160851	0.04899	0.0332648	22.2143181

cell								2
SSC-A on HLA DR+ CD4+ T cell	rs12482528	T	C	-0.2044	0.00889346	0.0433	0.0324766	28.0130972
SSC-A on HLA DR+ CD4+ T cell	rs3115859	A	G	-0.184	0.0032123	0.0376	0.0250791	23.94748755
SSC-A on HLA DR+ CD4+ T cell	rs138293681	T	G	-0.4259	-0.000956347	0.09115	0.0955273	21.83242285
SSC-A on HLA DR+ CD4+ T cell	rs6697448	A	C	-0.1533	-0.00749268	0.03184	0.0255319	23.18132157
SSC-A on HLA DR+ CD4+ T cell	rs4686701	A	G	-0.1248	-0.00616971	0.02639	0.0204947	26.62406545
SSC-A on HLA DR+ CD4+ T cell	rs4326672	C	T	0.1729	0.014699	0.03621	0.0322307	22.79989972
SSC-A on HLA DR+ CD4+ T cell	rs4941916	A	G	0.3194	0.0348624	0.07189	0.0868516	19.7393504
SSC-A on HLA DR+ CD4+ T cell	rs11910199	A	G	-0.1975	-0.0600085	0.04417	0.0439408	19.99306608

CD4RA on Terminally Differentiated CD4+ T cell	rs181435089	T	C	-0.4158	0.350319	0.08674	0.169451	22.97894758
CD4RA on Terminally Differentiated CD4+ T cell	rs4670907	T	C	-0.2086	0.0907225	0.04381	0.0374067	22.67159641
CD4RA on Terminally Differentiated CD4+ T cell	rs13269202	C	G	-0.204	0.0248933	0.04559	0.0244647	20.02263131
CD4RA on Terminally Differentiated CD4+ T cell	rs78528853	G	A	0.4624	-0.0516192	0.1037	0.0539571	19.8828272
CD4RA on Terminally Differentiated CD4+ T cell	rs76295302	C	G	0.2711	-0.0238161	0.06061	0.0394477	20.00647
CD4RA on Terminally Differentiated CD4+ T cell	rs1861266	C	A	0.1564	-0.0101676	0.03457	0.0217592	20.46796843
CD4RA on Terminally Differentiated CD4+ T cell	rs10815513	A	G	-0.1382	-0.00204247	0.03076	0.0246681	20.18568184
CD4RA on Terminally Differentiated CD4+ T cell	rs4261817	C	T	0.1204	0.00195388	0.02699	0.0198695	19.89973095
CD4RA on Terminally	rs4974277	T	A	0.3207	0.00543064	0.06869	0.0343686	21.7977108

Differentiated CD4+ T cell								4
CD4RA on Terminally Differentiated CD4+ T cell	rs147201746	A	G	-0.6008	-0.0280833	0.1219	0.0576174	24.29139392
CD4RA on Terminally Differentiated CD4+ T cell	rs931816	G	A	0.1874	0.0136724	0.04181	0.0259988	20.08994917
CD4RA on Terminally Differentiated CD4+ T cell	rs111916169	T	A	0.6182	0.0493648	0.1318	0.0665823	22.00022796
CD4RA on Terminally Differentiated CD4+ T cell	rs1504496	A	G	0.3839	0.0389232	0.08369	0.0301704	21.0421004
CD4RA on Terminally Differentiated CD4+ T cell	rs1328601	C	T	-0.5667	-0.0575607	0.1272	0.0486538	19.84870617
CD4RA on Terminally Differentiated CD4+ T cell	rs11652274	A	G	-0.1887	-0.0193813	0.04162	0.0245445	20.5560492
CD4RA on Terminally Differentiated CD4+ T cell	rs7559619	G	A	-0.2144	-0.0225134	0.02871	0.0200173	54.45011707
CD4RA on Terminally Differentiated CD4+ T cell	rs13292669	C	T	-0.1525	-0.0171961	0.03426	0.0254412	19.81367204

CD4RA on Terminally Differentiated CD4+ T cell	rs7894393	C	T	0.1644	0.0222136	0.03689	0.0262028	19.86032339
CD4RA on Terminally Differentiated CD4+ T cell	rs17777079	T	A	0.3498	0.0513178	0.0733	0.0682838	22.77359857
CD4RA on Terminally Differentiated CD4+ T cell	rs10114587	G	A	-0.2921	-0.0437745	0.06387	0.0606886	20.91554963
CD4RA on Terminally Differentiated CD4+ T cell	rs78948498	G	C	0.1761	0.0391256	0.03794	0.0277488	21.54388682
CD4RA on Terminally Differentiated CD4+ T cell	rs822833	T	C	-0.1386	-0.0401863	0.02992	0.0208109	21.45869377
CD4RA on Terminally Differentiated CD4+ T cell	rs2188219	C	G	-0.1239	-0.0392793	0.02773	0.0204368	19.96378452
CD4RA on Terminally Differentiated CD4+ T cell	rs929785	C	T	0.1273	0.0471436	0.02819	0.0245962	20.39232085
HLA DR on CD33+ HLA DR+ CD14dim	rs7667073	A	G	0.184	-0.0325152	0.04018	0.0221137	20.9708378
HLA DR on CD33+ HLA DR+	rs6912516	T	C	0.5936	-0.054991	0.1328	0.0801429	19.9798229

CD14dim								1
HLA DR on CD33+ HLA DR+ CD14dim	rs34813869	G	A	0.229	-0.0194249	0.04567	0.0205441	25.14252794
HLA DR on CD33+ HLA DR+ CD14dim	rs613165	A	G	-0.2784	0.00766986	0.04395	0.0215747	40.12552272
HLA DR on CD33+ HLA DR+ CD14dim	rs2094597	T	C	-0.1688	-0.00548878	0.03709	0.0199782	20.71243783
HLA DR on CD33+ HLA DR+ CD14dim	rs12987451	G	A	-0.1883	-0.0106767	0.04167	0.0215759	20.41990133
HLA DR on CD33+ HLA DR+ CD14dim	rs9270588	T	C	-0.4302	-0.0274552	0.04022	0.0197837	368.180656
HLA DR on CD33+ HLA DR+ CD14dim	rs2115412	C	G	0.3923	0.0282855	0.07994	0.0424683	24.08287483
HLA DR on CD33+ HLA DR+ CD14dim	rs7478464	G	A	0.2261	0.0184951	0.04973	0.0281785	20.67112943
HLA DR on CD33+ HLA DR+ CD14dim	rs393073	T	A	-0.1732	-0.0160065	0.03878	0.0209638	19.94711842

HLA DR on CD33+ HLA DR+ CD14dim	rs146788953	A	G	-0.455	-0.0496576	0.1004	0.0402483	22.90646271
HLA DR on CD33+ HLA DR+ CD14dim	rs9846278	T	C	-0.3738	-0.048535	0.08102	0.0420176	21.28600287
HLA DR on CD33+ HLA DR+ CD14dim	rs9346529	A	G	-0.1648	-0.0226216	0.03674	0.0201511	20.12037685
HLA DR on CD33+ HLA DR+ CD14dim	rs112878149	C	G	-0.4619	-0.0741806	0.09902	0.0494142	21.75955856
HLA DR on CD33+ HLA DR+ CD14dim	rs79485969	T	C	0.3333	0.0556324	0.074	0.0272688	20.28650292
HLA DR on CD33+ HLA DR+ CD14dim	rs111404935	C	T	-0.4016	-0.0851805	0.07817	0.0576796	26.39411985
HLA DR on CD33+ HLA DR+ CD14dim	rs142241717	A	T	0.5061	0.254931	0.107	0.145226	22.3720159
HLA DR on CD33+ HLA DR+ CD14dim	rs142186496	G	A	0.4579	0.372814	0.07698	0.328508	35.38225218

Note: SNPs: single nucleotide polymorphisms; SE:standard error;
