# Supplementary material for: Causal effect of tea consumption on the increased risk of puerperal sepsis and the mediation effect of CD25 on IgD- CD38-B cell: A Mendelian randomization analysis
Source: Medicine (Baltimore). 2025 Oct 24;104(43):e44721. doi: 10.1097/MD.0000000000044721 (PMC12558255; doi:10.1097/MD.0000000000044721)
Supplement: Supplementary file 2 [file medi-104-e44721-s002.pdf]

Figure S1 Scatter plot for two-step Mendelian randomization (MR) analysis for 26 immunological traits A: scatter plot for the causal effect of tea consumption on 26 immunological traits; B: scatter plot for the causal effect of 26 immunological traits on puerperal sepsis.

Figure S2 Forest plot for each SNP of two-step Mendelian randomization (MR) analysis for 26 immunological traits A: each SNP for the causal effect of tea consumption on 26 immunological traits; B: each SNP for the causal effect of 26 immunological traits on puerperal sepsis.

Figure S3 Leave-One-Out method for two-step Mendelian randomization (MR) analysis for 26 immunological traits A: Leave-One-Out method for the causal effect of tea consumption on 26 immunological traits; B: Leave-One-Out method for the causal effect of 26 immunological traits on puerperal sepsis.

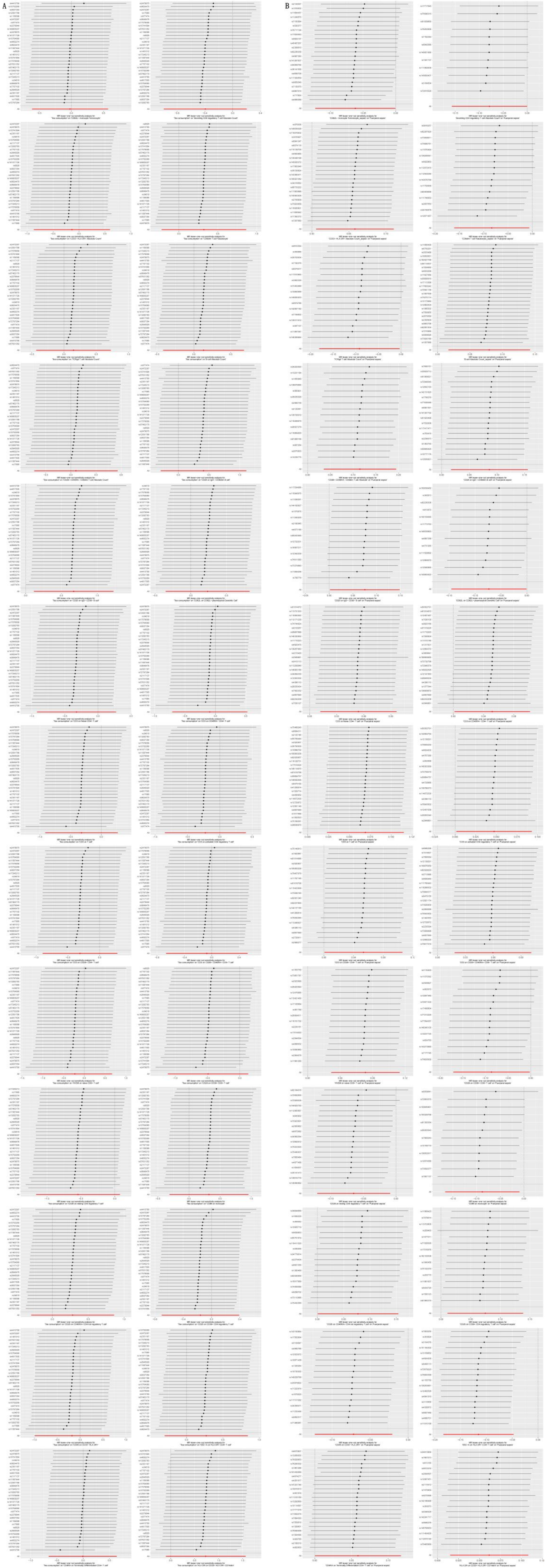
A
B

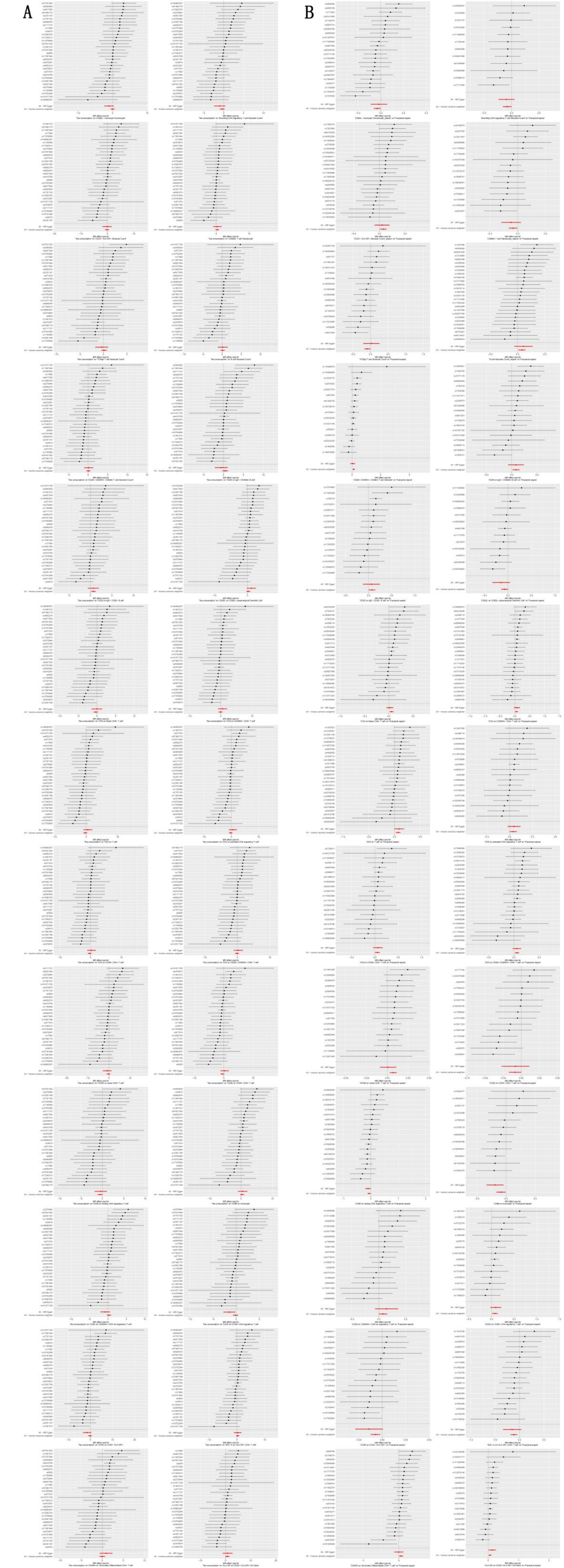
A
B

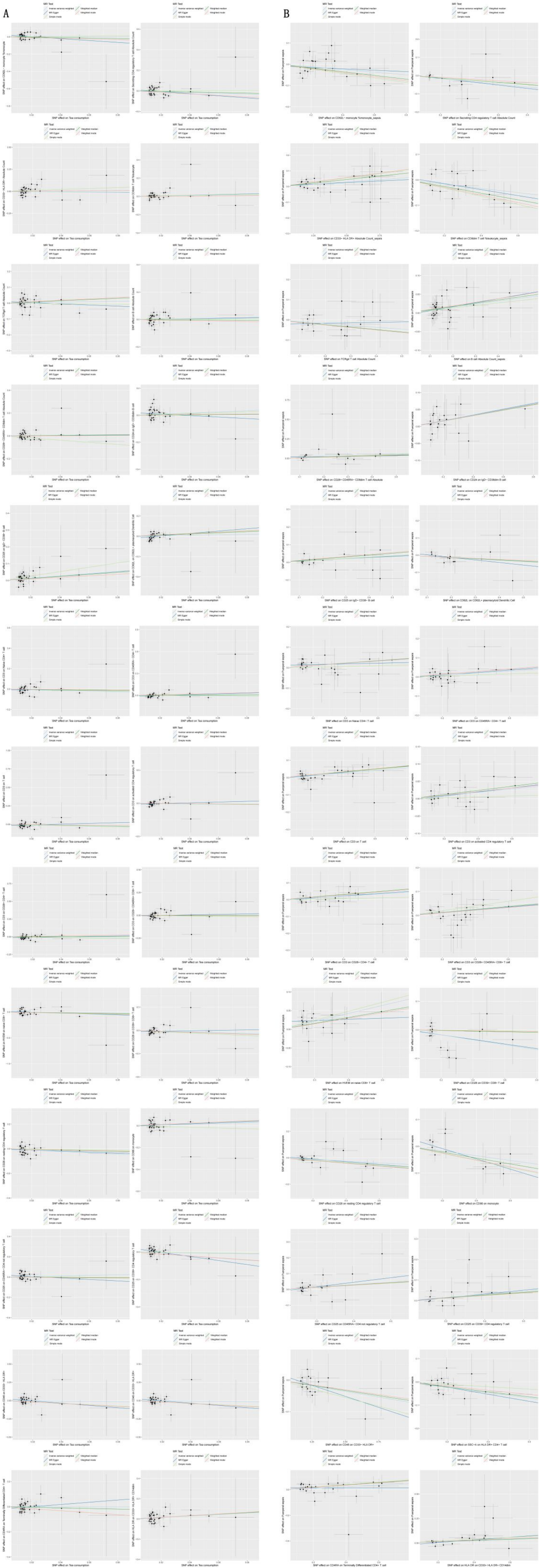
A
B
